# Freshwater monitoring across the globe: the role of citizen science within the European Water Framework Directive (WFD) and the United Nations Sustainable Development Goals (SDGs), and opportunities to incentivize the collaboration with environmental regulators

**DOI:** 10.12688/openreseurope.19162.3

**Published:** 2025-08-20

**Authors:** Bruna Gumiero, Leonardo Veronesi, Luisa Galgani, Riccardo Gaetano Cirrone, Alessio Corsi, Andrea Tafi, Steven Arthur Loiselle

**Affiliations:** 1 CSGI - Consorzio Interuniversitario per lo Sviluppo dei Sistemi a Grande Interfase, Via della Lastruccia 3, 50019 Zona Osmannoro Firenze, Italy; 2 Università degli Studi di Bologna, Bologna, Italy; 3 National Biodiversity Future Center, NBFC, Piazza Marina 61, 90133 Palermo, Italy; 4 European Citizen Science Association - ECSA, c/o Museum für Naturkunde, Invalidenstraße 43, 10115, Berlin, Germany; 5 Dipartimento di Biotecnologie, Chimica e Farmacia - DBCF, Università degli Studi di Siena, Via Aldo Moro 2, Siena, 53100, Italy; 6 Consiglio Nazionale delle Ricerche (CNR), Istituto di Scienze Marine (ISMAR), Forte Santa Teresa, Pozzuolo di Lerici, 19032 Lerici (SP), Italy

**Keywords:** citizen science, freshwater ecosystems, Water Framework Directive, Sustainable Development Goals

## Abstract

Citizen science plays a crucial role in advancing the objectives of the European Union’s Water Framework Directive (WFD) and the United Nations Sustainable Development Goals (SDGs). Among the key strengths of citizen science is that it fills information gaps in the management and observation of aquatic ecosystems, especially small rivers that often lack national and sub-national agency monitoring. The present study explores opportunities and challenges of integrating citizen science data with those of Environmental Agencies. The current state of the art is discussed through an analysis of 85 publications dealing with freshwater citizen science, finding that 34 of the ones individuated actually use citizen-science generated data. These 34 studies were analysed in more details focusing on data quality and geographical distribution. Findings highlight that citizen-generated data reach an accuracy between 70% and 90% when compared to laboratory values, but despite this outcome there is often lack of trust in citizen science data and processes. This is reflected in a limited involvement with policymakers and regulatory agencies. The present publication highlights good practices, challenges and opportunities for collaboration with environmental agencies, giving examples of some projects to address the WFD and increase the impact of freshwater citizen science.

## Introduction: empowering communities to reach good environmental status

Freshwater ecosystems are facing an ever-increasing range of chemical and physical stressors, often connected to global and regional changes in population growth, agricultural intensification, urbanisation, limited wastewater treatment and climate change ( van Rees *et al.*, 2021). Nutrient enrichment is a common challenge across the globe, and is amplified by changes in land use, river morphology and climate. Eutrophication has led to the loss of river and lake functioning as well as to the increased frequency of harmful algal blooms (HABs) ( Birk *et al.*, 2020; Reid *et al.*, 2019). These combined stressors pose at risk the health of the ecosystems themselves and the capacity of the ecosystems to provide services to mankind. Effective and efficient monitoring is paramount to document rapid ecosystem changes, but often regulatory agencies and research institutions lack the power and the resources to cover sufficiently large temporal and spatial dynamics to address ecosystems’ alterations.

**Figure 1.**
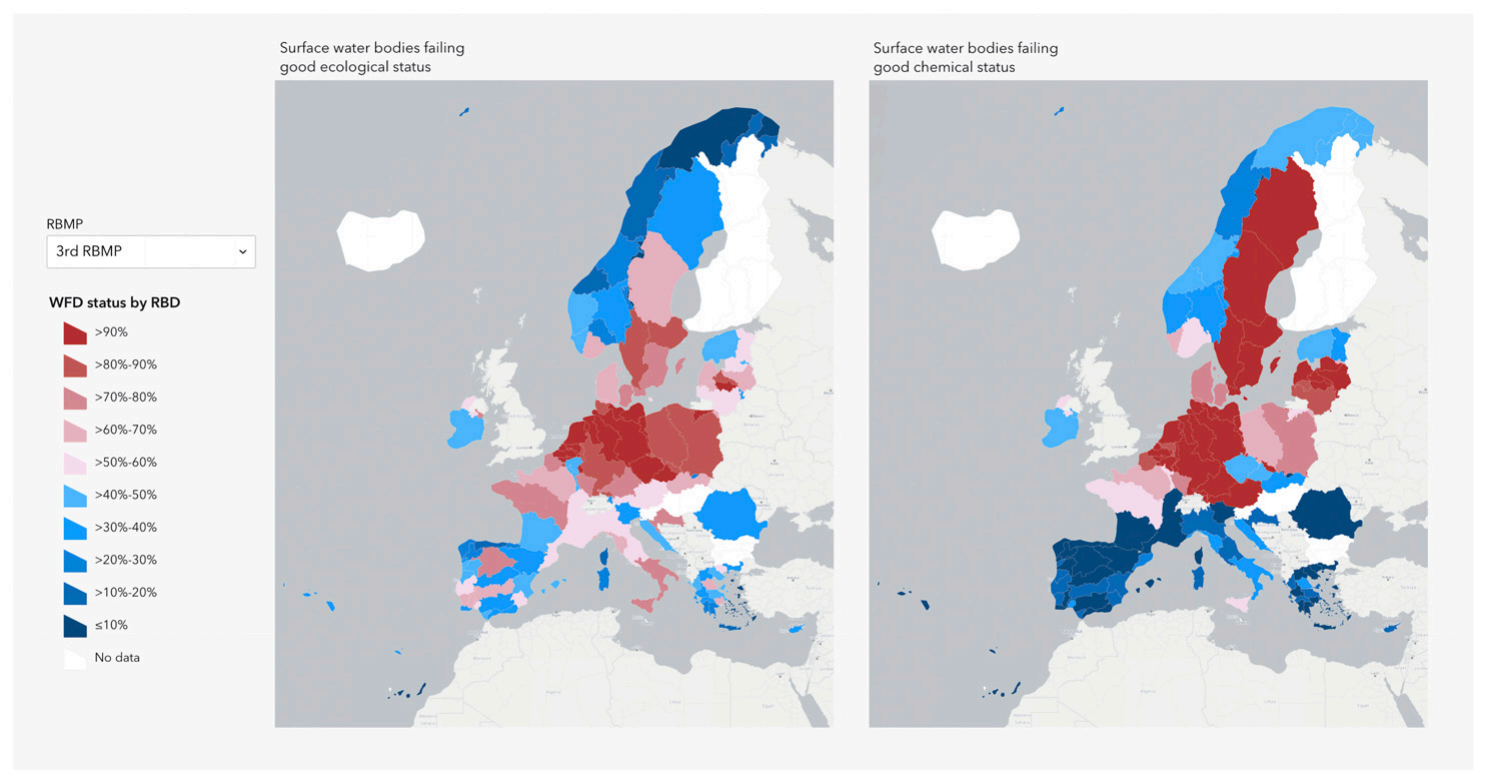
Proportion of European surface water bodies failing to achive good chemical and ecological status, at River Basin District (RBD) level, for the 3rd cycle of the Water Framework Directive (WFD) - River Basin Management Plan (RBMP)(2021). https://water.europa.eu/freshwater/europe-freshwater/maps/chemical-status-map-by-rbd and https://water.europa.eu/freshwater/europe-freshwater/maps/ecological-status-map-by-rbd.

In Europe, the European Water Framework Directive (WFD) requires all EU member countries to monitor the “ecological status” of surface waters in a consistent and strategic manner ( Carvalho *et al.*, 2019). The WFD takes into consideration inland water bodies, groundwaters, as well as surface coastal and transitional waters, setting a goal to reach “good status, and to prevent deterioration” of all water bodies in the European Union and Norway with River Basin Management Plans by member states. The objectives were set in 2000 when the WFD was implemented as the main law for water protection and should be achieved by 2027. Every River Basin Management Plan includes the assessment of water bodies’ ecological and chemical status within the river basin, the pressures all aquatic ecosystems are undergoing, as well as relevant plans towards achieving good status. The good chemical and ecological status are assessed by biological, hydromorphological, physico-chemical properties, and chemical pollutants’ concentration. The WFD currently covers more than 146500 surface water ecosystems and 15000 groundwater bodies in the EU and Norway (WISE website). As the 2027 deadline approaches, the situation in nearly all member states is far from the prospected objective ( Figure 1).

To assess the status of freshwaters on a global level, common strategies have been put forward within the Sustainable Development Goals framework (SDGs), but national monitoring networks in many regions are either inadequately resourced, poorly coordinated, or non-existent ( UN-Water, 2018). While freshwater is recognized as a global, essential good within the SDGs framework, it is addressed by specific goals and sub-targets. SDG nr. 6 focuses on “ensuring access to clean water and sanitation for all”, and encompasses specific sub-targets: 6.3, specifically aiming to “improve water quality by reducing pollution, eliminating dumping and minimising release of hazardous chemicals and materials, halving the proportion of untreated wastewater and substantially increasing recycling and safe reuse globally” by 2030, 6.5, to “implement integrated water resources management at all levels, including through transboundary cooperation as appropriate” and target 6.b, to “support and strengthen the participation of local communities in improving water and sanitation management”. Additionally, SDG 15 aims to “protect, restore and promote sustainable use of terrestrial ecosystems (including freshwater ecosystems), sustainably manage forests, combat desertification, and halt and reverse land degradation and halt biodiversity loss”. In order to track the progress towards the 6.3 SDG target in particular, each country adhering to the SDGs framework is required to report their advancements using the indicator 6.3.2 which reports “the proportion of bodies of water with good ambient water quality, as per national and/or subnational water quality standards and based on measurements of five water quality parameters (Oxygen, Nitrate, Phosphorus, Salinity, Acidification) that inform on the most common pressures on water quality at the global level” (SDG 6.3.2 website). According to the SDGs indicator 6.3.2, in the years 2017–2023 56% of the world’s monitored water bodies was reported having good ambient water quality. This information however bears a high amount of unavailable data because of a reduced monitoring capacity in low-income countries. Since the health status of freshwater ecosystems in these regions is not routinely surveyed, and clean water is not regularly reported, this lack of information poses over 3 billion people at risk (SDG indicator 6.3.2 website, United Nations Environment Programme, 2021).

In the necessity to improve data acquisition, increase spatial and temporal coverage and fill knowledge gaps on global freshwaters’ health and ecological status, aligning with regional (WFD) and global (SDGs) policy frameworks and requirements, citizen science becomes a key approach. Citizen science, that is, any activity that involves the public in scientific research, bringing together science, policy and society in the creation of scientific evidence and knowledge, offers a valid complementary research tool as several parameters can be monitored in freshwater environments. While this methodology helps agencies fill in data knowledge gaps, at the same time, it proves an opportunity to strengthen scientific and environmental literacy in support of freshwaters ecosystems’ management. A number of studies have in fact suggested that citizen science can assist national and subnational objectives related to the WFD ( Carvalho *et al.*, 2019; European Commission, Directorate-General for Environment, 2018) as well as the SDGs ( Biraghi *et al.*, 2022; Fraisl *et al.*, 2020; Fritz *et al.*, 2019; Hegarty *et al.*, 2021; Quinlivan *et al.*, 2020; Venkatesh & Velkennedy, 2023).

**Figure 2.**
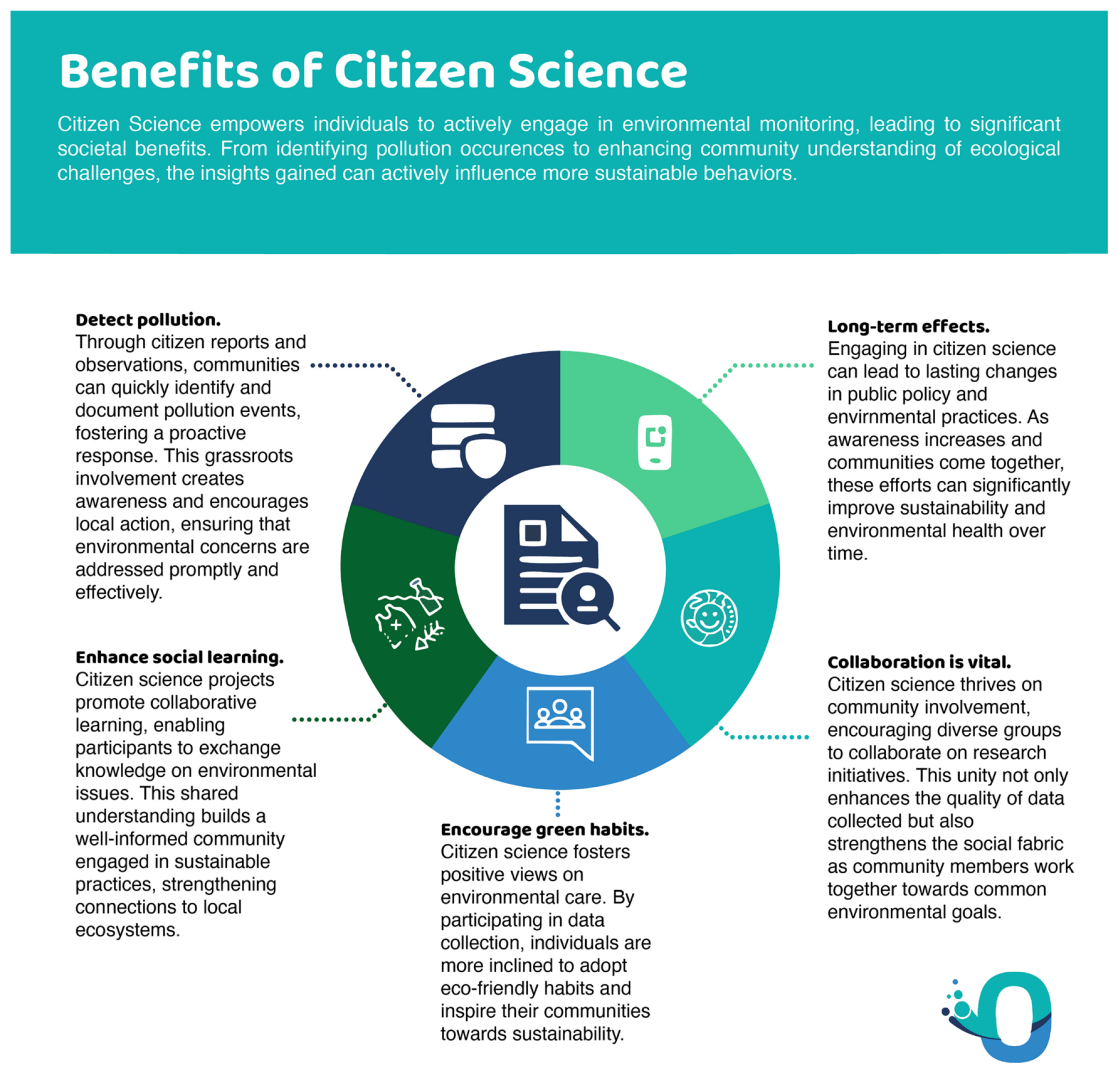
Positive impacts of citizen science. Citizen scientists can quickly identify and document pollution events, while gaining awareness and environmental stewardship. Sustained engagement and collaboration among diverse groups can lead to policy interventions towards sustainability. Citizen science also promotes social community learning: the shared information and knowledge generated among participants fosters people’s connection to local ecosystems.

Citizen science can create a positive environmental impact across a range of related areas, with studies showing that it also has the potential to identify pollution events ( Collins *et al.*, 2023), improving social learning around environmental issues, and contributing to shaping attitudes and behaviours towards a more sustainable lifestyle, if the project is designed adequately ( Fielke *et al.*, 2022; Jørgensen & Jørgensen, 2021; van Noordwijk *et al.*, 2021) ( Figure 2). While this presents as a fast-growing opportunity to gather information and foster public participation in environmental compliance, citizen science needs specific guidelines and standardization methods to support environmental monitoring and create comparable results across countries and similar objectives. At the same time citizen science needs to encompass a strong public participation and social implications to create a meaningful impact, but in some WFD related projects the actual citizen participation has been so far limited ( Rimmert *et al.*, 2020) or challenging ( van der Heijden & ten Heuvelhof, 2012). Citizen science thus represents a noteworthy opportunity to complement standard monitoring frameworks in Europe and across the globe; however, it needs standardization to acquire data reliability and comparability, as well as an alignment with open and FAIR (Findable, Accessible, Interoperable, and Reusable) data principles to deliver lasting value and drive substantial change.

Given the rising recognition of environmental literacy and citizens’ empowerment to address climate and environmental challenges, and foster communities’ resilience facing pollution and the increasing frequency of environmental extreme events, the present open letter explores citizen science-based projects monitoring freshwater quality. This exercise is based on an assessment of the current available information, projects and papers that focus on specific aspects of citizen science in freshwater environments with the aim to identify challenges and opportunities, as well as the potential for collaboration with national Environmental Agencies to ensure a sustained engagement and a longer-term commitment, aligning with regional and international policy frameworks. Ultimately, based on the current knowledge, it provides suggestions to overcome hurdles in upcoming research programmes and activities.

## State of the art

This study is based on an analysis of existing literature describing citizen science projects in freshwater environments; papers were searched in Scopus and Google Scholar in a snowball quest according to a set of keywords that ranged from the type of scientific data used to relevant directives, most relevant projects, terminology linked to the river ecosystem, and terminology linked to citizen science ( Table 1). The ‘snowball quest’ refers to the practice of identifying a main source – a topic-relevant publication with clear impact (i.e. a high number of citations) – and go through the references present in that publication looking for more similar topic-relevant publications. Alongside this search method, different types of keywords were mixed when searching in Scopus and Google Scholar, usually one keyword concerning the scientific aspects with one or more keywords concerning citizen science; the “citizen science” keyword was the most commonly used, as it is quite often found in publications where the approach is utilised. The keywords were chosen according to the following criteria: they stem from expert knowledge of the authors in the field of freshwater citizen science and are centered around the European context, both for the relevant directives and the projects surveyed.

**Table 1.** List of keywords used by topic for the Scopus and Google Scholar search of papers for citizen science projects in freshwater environments.

*Topic*	*Keywords*
**Scientific parameters**	Macrobenthos; turbidity; nitrates and phosphates concentrations; geomorphology; fishes; plastic
**Directives**	WFD; SDGs
**Most relevant European projects**	RiuNet; Riverfly; Flow; FreshWater Watch; Merlin
**Citizen science**	Co-design; impact; behaviour; experimental design; early warning process; Environmental Agencies collaboration; stakeholder involvement

**Figure 3.**
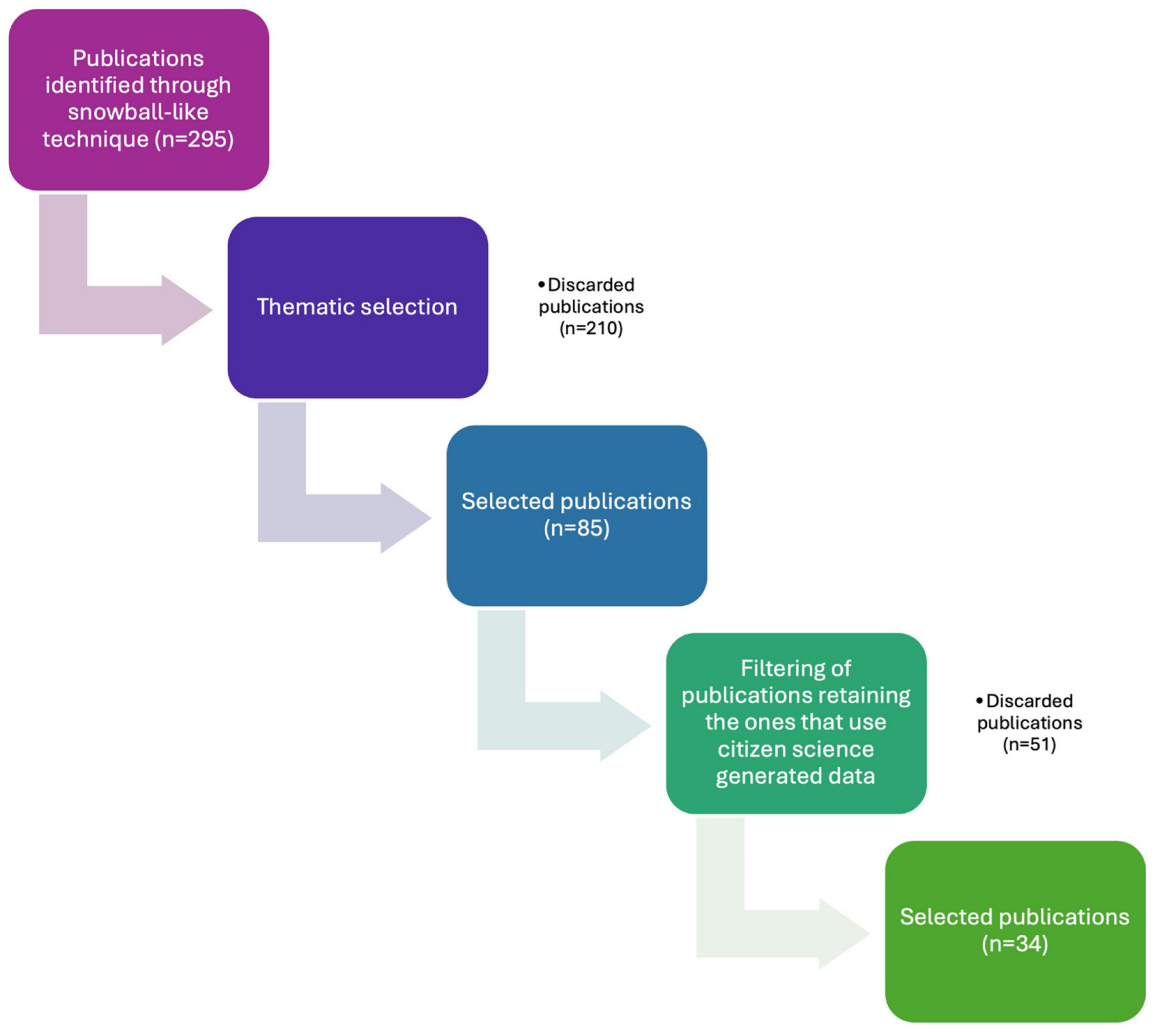
Flowchart of the publications' selection process.

We identified 295 papers that address citizen science to some extent, of which 85 were thematically selected by filtering them by topic: the main themes of the publications were screened by reading their abstracts, set of keywords, and results looking for direct mentions of the topics we were mainly interested in, namely WFD, SDGs, and Environmental Agencies collaboration. We ended up identifying other relevant topics too, based on our interests and what emerged from the literature review as relevant links to our main topics. Such extra topics were: , Social perspective, Emerging Pollutants, Ecopsychology and Other ( Figure 3).

**Figure 4.**
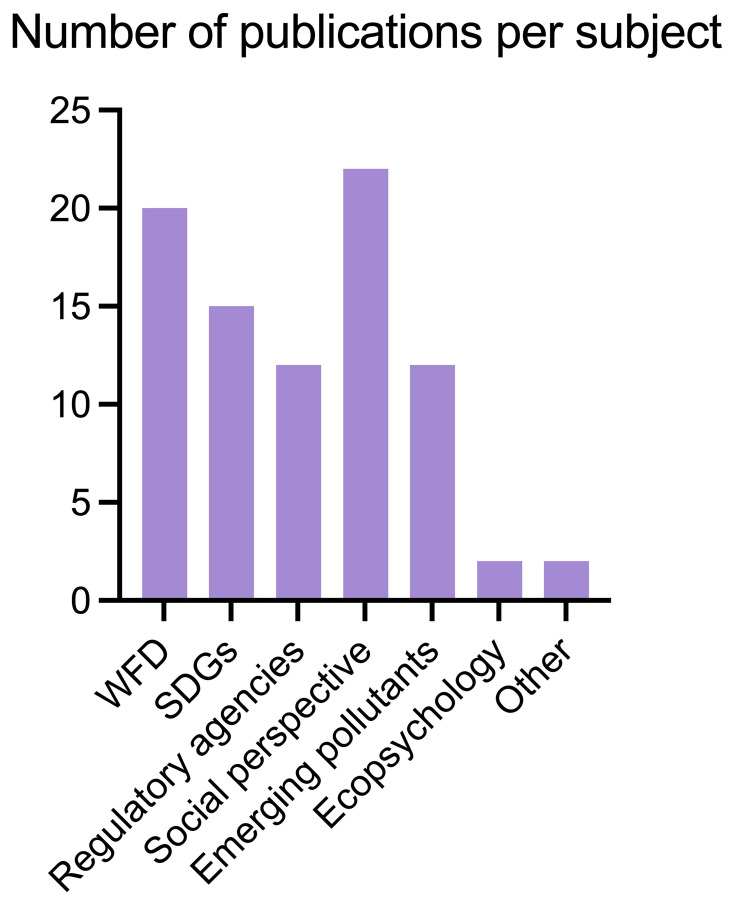
Publications found per subject area (n =85).

**Figure 5.**
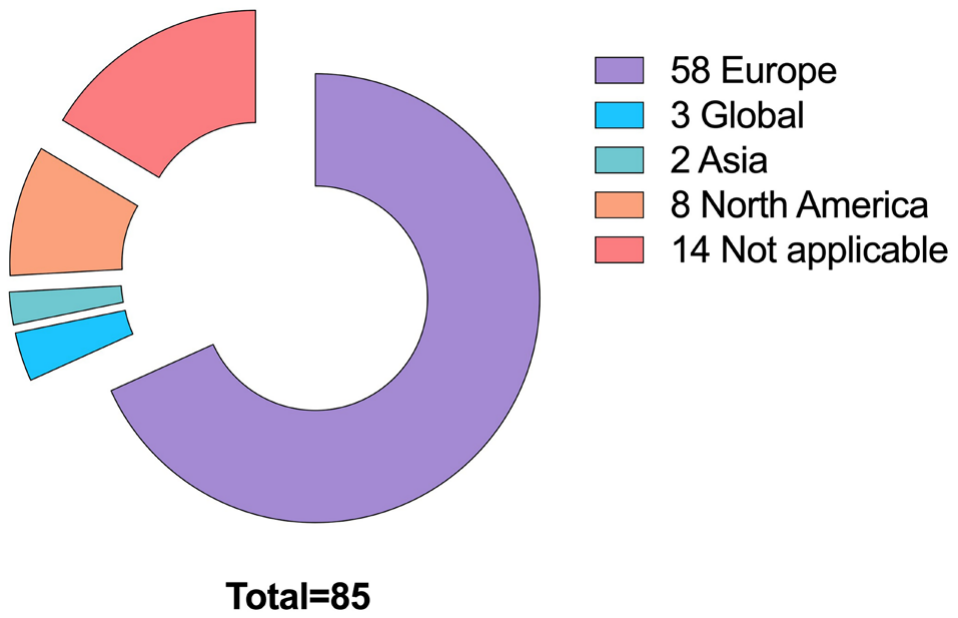
Geographical distribution of publications (n=85).

More specifically, we selected 17 papers linked to WFD, 13 for SDGs, and 55 for other themes such as Regulatory Agencies collaboration, social and political perspective and more ( Figure 4). We also looked at the geographical distribution of these publications ( Figure 5). Of the 85 selected papers, 34 clearly reported citizen-science generated data and results and were therefore analysed based on this. They were deemed to represent citizen-science generated data as citizen science methodologies, as well as the involvement of citizens were clearly stated in the publications; furthermore, they followed ECSA’s 10 principles of citizen science to some extent. Specifically, the most recurrent principles were those of impact – scientific and social, involvement of citizens in different phases of the research, and scientific outcome. Citizens were also frequently acknowledged for their contribution in the research, figuring as co-authors or cited in the publications in many instances. Finally, many authors made their data public in an open-access manner ( ECSA, 2015).

The publications which were not considered strictly citizen science were mostly literature reviews or opinion papers, nevertheless considered useful as they treated the subjects of interest, but not directly using citizen science generated data for their output. These publications were therefore not thoroughly analysed, but noted nonetheless.

## The backbones of citizen science

### Approaches, parameters, and data quality

We are here focusing on projects that can rely on citizens both for the gathering and the analysis of data, where the in-situ data collection and analysis are feasible. The 34 studies analysed in detail show that data gathered by citizen scientists reach an accuracy between 70% to 90% of laboratory values, and more than sufficient to complement agency monitoring ( Breuer *et al.*, 2015; de Sherbinin *et al.*, 2021; Fritz *et al.*, 2019; Pinto *et al.*, 2020; Quinlivan *et al.*, 2020; Stankiewicz *et al.*, 2023; von Gonner *et al.*, 2023). Our screening highlighted that an increasing number of publications report the use of citizen science acquired data for hypothesis testing and complementary monitoring of water quality in European freshwater ecosystems.

**Figure 6.**
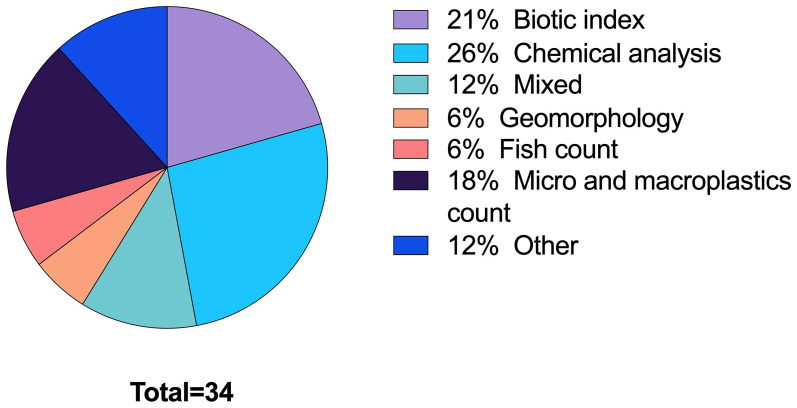
Analytical approaches in the publications identified (n=34).

In all the publications screened that addressed citizen science (n = 85) the most common parameters studied were water chemistry and macrobenthos. In the 34 selected publications, mixed parameters refer to more than one of the listed indicators, among which macrobenthos was always present. The ‘other’ category comprises water quality analysed through the presence of underwater vegetation, the turbidity level using a Secchi disk, and other less common approaches ( Figure 6). Among the latter are fauna and litter sightings used as a proxy for water quality ( Biraghi *et al*., 2022) and qualitative analytical methods – in the form of semi-structured interviews – to harvest the local population knowledge and perception of the declining presence of fish in rivers in the last decades ( Stenekes *et al*., 2020).

As quality control is a key discriminant in the use and reputation of citizen science data, we looked for the presence of either direct comparison to professional gathered data, professional screening of the obtained data, or validated techniques of data gathering - such as the FreshWater Watch initiative (FreshWater Watch website). It has been reported that quite often side by side measurements on a fixed number of samples allow to compare professional gathered data and volunteer gathered data to evaluate the precision and accuracy of the latter ( von Gonner *et al.*, 2023). Other approaches involve statistical comparisons between expert and citizen collected data to show that indeed citizen science data is good enough ( Hadj-Hammou *et al.*, 2017).

**Figure 7.**
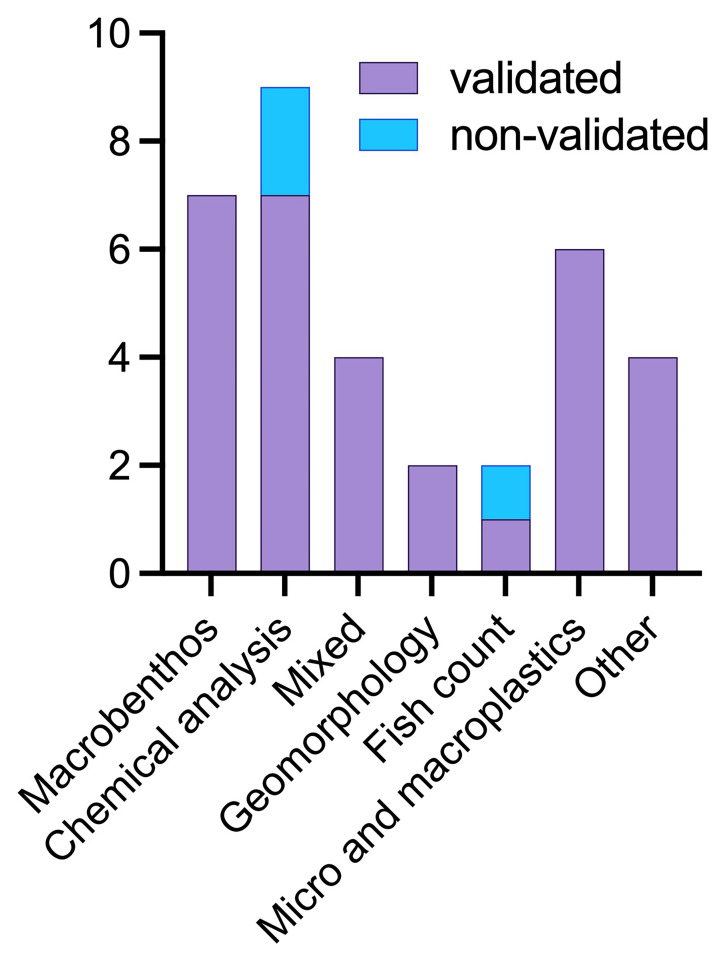
Number of data-validated and non-validated papers per methodology applied (n = 34).

In the monitoring of freshwater ecosystems, chemical approaches to determine water quality and presence and identification of macrobenthos communities are widely used robust methods, addressing the WFD set of standards to define the good chemical and ecological status of freshwater water bodies. Both approaches can be done in-situ by trained citizen scientists and therefore, are being addressed by several initiatives, helping regulatory agencies and research institutions to cover monitoring gaps. From our analysis of the selected 34 studies, it emerged that most of the projects have data validation techniques in place (n = 31), either with cross validation with laboratories, or statistical analyses ( Figure 7).

Implementing cross-validation methods can enhance the reliability of the findings. By comparing citizen data against established benchmarks or expert assessments, we can strengthen credibility and ensure that contributions are recognized as valuable and accurate.

Some contaminants of emerging concern (CECs) like plastic and microplastics are also suitable parameters for in situ monitoring by trained citizens and volunteers because these compounds are relatively stable and do not need special laboratory treatment for immediate visualisation and analysis ( Vasantha Raman *et al.*, 2023). So far, most citizen science efforts on plastic and microplastics have concentrated on marine systems ( Blettler *et al.*, 2018), especially on beaches, with a minor number of initiatives focused on freshwater environments. However, harmonization of data and methods to monitor plastic and microplastic pollution is still a bias in professional scientific research. Therefore, citizen science projects may be properly incorporated to complement data gaps on a European (WFD) and global (SDGs) scale when a standardization framework is in place, allowing the intercomparison of information and data exchange.

### Quality of the publications analysed

While the selection of publications was based on keywords by topics, the ranking of the publications to assess their “quality” was instead based on a series of internally discussed criteria. It should be noted that this purely qualitative approach is subjective and should be seen as a non-standardized method to look at paper quality, which of course can introduce biases in the analysis. With this limitation in mind, we tried to follow a specific path where each team member’ choices were re-validated and discussed in a group. This exercise was developed in the following way: when one person did the ranking, an internal validation by two more members of the team looking at such rankings followed, to finally build an aggregate scale in a group discussion.

The publications were sorted in a five-point scale ranging from “very good” to “very weak”, which we assigned for each of five different categories: a) citizen involvement; b) scientific practices; c) approach originality; d) associated impact; e) clarity of practices.

a) Citizens' involvement refers to the type of involvement, whether they were involved in multiple stages of the project or not.
b) Scientific practices look at if the scientific practices were grounded in the scientific literature, and if the volunteers were autonomous in their sampling and analysis, or a lab was needed and thus citizens were merely sampling.
c) Approach originality tries to capture if the approach is innovative or if it reflects other similar studies.
d) Associated impact tries to understand if there was any positive associated impact stemming from the project.
e) Clarity of practices, finally, looks at whether the research practices involved were clearly described: e.g. was the volunteers training documented, who was involved, in what phases, etc.

**Figure 8.**
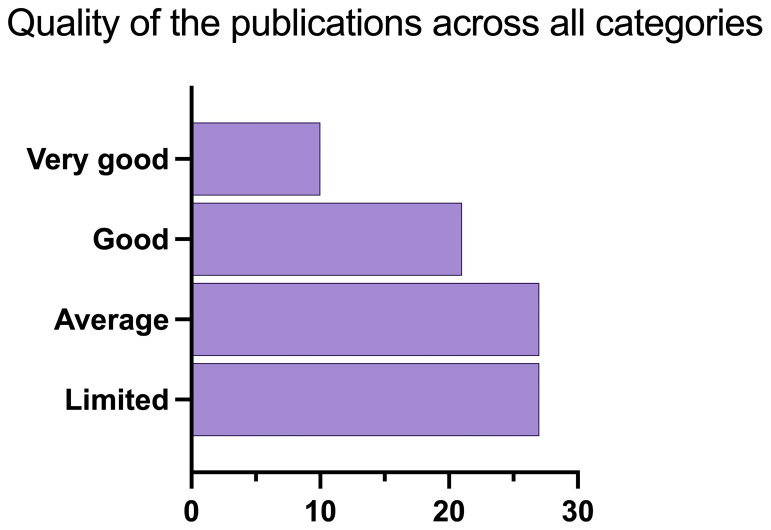
Quality of papers using the aggregate scale (n=85).

**Figure 9.**
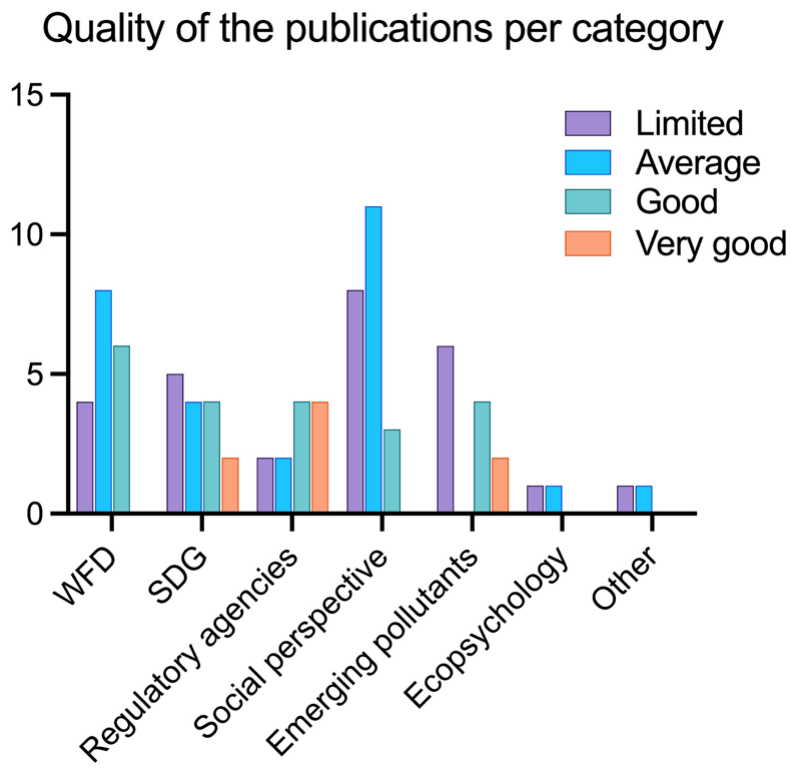
Quality of publications per category using the aggregate scale (n = 85).

To build the aggregate scale, each “very good” was assigned 2 points, “good” was assigned 1 point, while “average”, “weak” and “very weak” were assigned 0 points respectively. The points per each category were then summed up per each publication. With a final score of 1, papers were labelled as “limited”, with a score of 2 “average”, with a score of 3–4 “good”, and with a score of 5 or higher “very good” ( Figure 8, Figure 9).

Figure 8 shows the overall publications’ quality, that includes all papers (n = 85). Most of the publications fall into the categories “limited” and “average”, while only 10 and just above 20 are good or very good.

Figure 9 shows the quality of papers per category, namely WFD, SDGs, Environmental Agencies, Social perspective, Emerging Pollutants, Ecopsychology and Other. Most papers that are labelled as “good” or “very good” fall into either WFD, SDGs, Regulatory Agencies or Emerging pollutants. This means that there are very few or none in the categories Social perspective, Ecopsychology, and Other.

The categories used to rank the publications’ quality are skewed towards the hard scientific approach, in other terms a publication (and project) that clearly reports citizens’ involvement and good scientific practices is favoured. Since publications on social perspectives and perceptions do not normally have citizens involved nor present a hard scientific approach of environmental monitoring, they heavily fall into the “average” and “limited” score.

Papers that deal with Regulatory Agencies collaboration are ranked quite high: this reflects our judgement on these publications as the collaboration with Regulatory Agencies, often not present in citizen science, was considered a positive trait and an effort worth pursuing.

### Social aspects of engagement and commitment over time in citizen science projects

In the 85 publications addressing citizen science in general, 24% of the initiatives individuated are long-term projects, 3% short-term projects, 5% blitzes. The vast majority, 68%, are projects in our opinion not properly structured as citizen science when comparing their design and workflow to the ten principles of citizen science delineated by the European Citizen Science Association ( ECSA, 2015), rather, they include papers like literature reviews and studies made only by experts. These ten principles aim to address good practice in citizen science: they try to create a consistent framework for the structure of citizen science projects across different disciplines and communities, addressing social and technical aspects of citizen science like volunteer engagement and retention, as well as data collection and transparency of the whole scientific process. In the duration of citizen science projects, the difference between blitz and short-term lies in the fact that blitz-type research is a one-time event comprising all fieldwork, while in short term projects more than one fieldwork event is held. Since long term projects are more cost and effort demanding, why are they often still chosen over short term ones? Apart from research objectives, from a social perspective long term projects tend to have a higher impact on participants and offer a higher potential to serve environmental policy making. In fact, it is widely reported that the desired impacts of citizen science on participants are social diffusion and behavioural change ( Church *et al.*, 2019), increased environmental awareness among the general public through citizen scientists social networks ( Johnson *et al.*, 2014), which are outcomes visible with time. The longer project duration can also support a stronger positive environmental change in terms of improved “(1) environmental management; (2) evidence for policy; (3) behavioural change; (4) social network championing; (5) political advocacy; and (6) community action” ( van Noordwijk *et al.*, 2021).

It is a common understanding among authors that for the success of citizen science initiatives, alongside scientific aspects of data trustworthiness and validation, a focused integration of both hard and social sciences is needed, but often lacking ( Nardi *et al.*, 2022; Newson, 2022). The call for the inclusion of different disciplines arises from the fact that social sciences are usually better equipped when dealing with engagement, impact assessment and participants’ motivations, by surveys or other methods ( Ballard *et al.*, 2017; Church *et al.*, 2019). The engagement in citizen science projects is an effective way to improve citizens’ awareness and aquatic literacy, thereby influencing behavioural change towards SDGs and good water quality of aquatic environments.

It is indeed well recognized that engaging the public is often challenging for professional scientists, with some categories of participants being overrepresented in citizen science projects ( Altman *et al.*, 2023; Haywood & Besley, 2014; Kaplan Mintz *et al.*, 2023), as high school and university students and people who are already part of environmental projects or environmentally engaged or literate. In freshwater environments, fishing communities, as well as members of local associations (e.g., local hiking groups, hobbyists or other “active citizenship” volunteers) are the people most involved in citizen science projects to date as these participants’ categories are easier to recruit and are already sensitive to environmental issues (for examples of participants, see the sample projects’ description in the section "Examples of best practices"). This results in a participation bias within most citizen science projects and the limited representation of marginalized parts of society.

**Figure 10.**
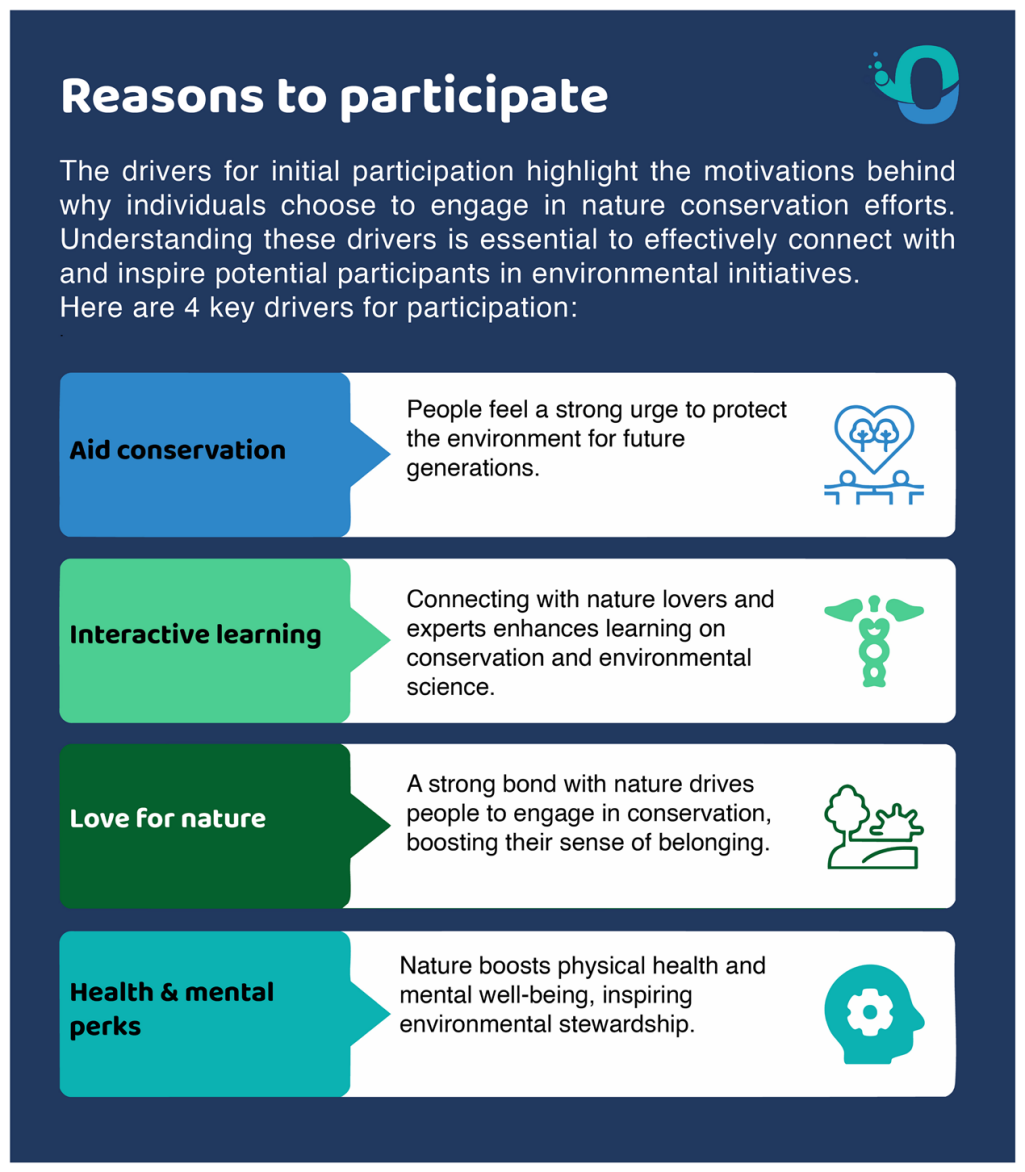
Drivers for initial participation in citizen science projects.

Several studies have focused on the motivational drivers of participants; why people want to participate in the projects and what keeps them engaged for extended periods ( Altman *et al.*, 2023; Church *et al.*, 2019; Kaplan Mintz *et al.*, 2023). These studies highlight the practices that have been successful at maintaining high participant retention rates, fundamental for ensuring project sustainability as well as improving data quality, typically higher when measurements are made by participants with longer experience. Drivers for initial participation include the desire to contribute to nature conservation and the possibility to learn through social interaction or interaction with professional scientists ( Kaplan Mintz *et al.*, 2023; Moshi *et al.*, 2023). More specifically to freshwater citizen science, positive feelings towards rivers, desire to learn about science and nature have been identified among the drivers for participation ( Church *et al.*, 2019). The beneficial physical and positive mental health effects from spending time in nature, explored in studies of *ecopsychology* should also be considered ( Cameron *et al.*, 2020; Roszak *et al.*, 1995) ( Figure 10).

**Figure 11.**
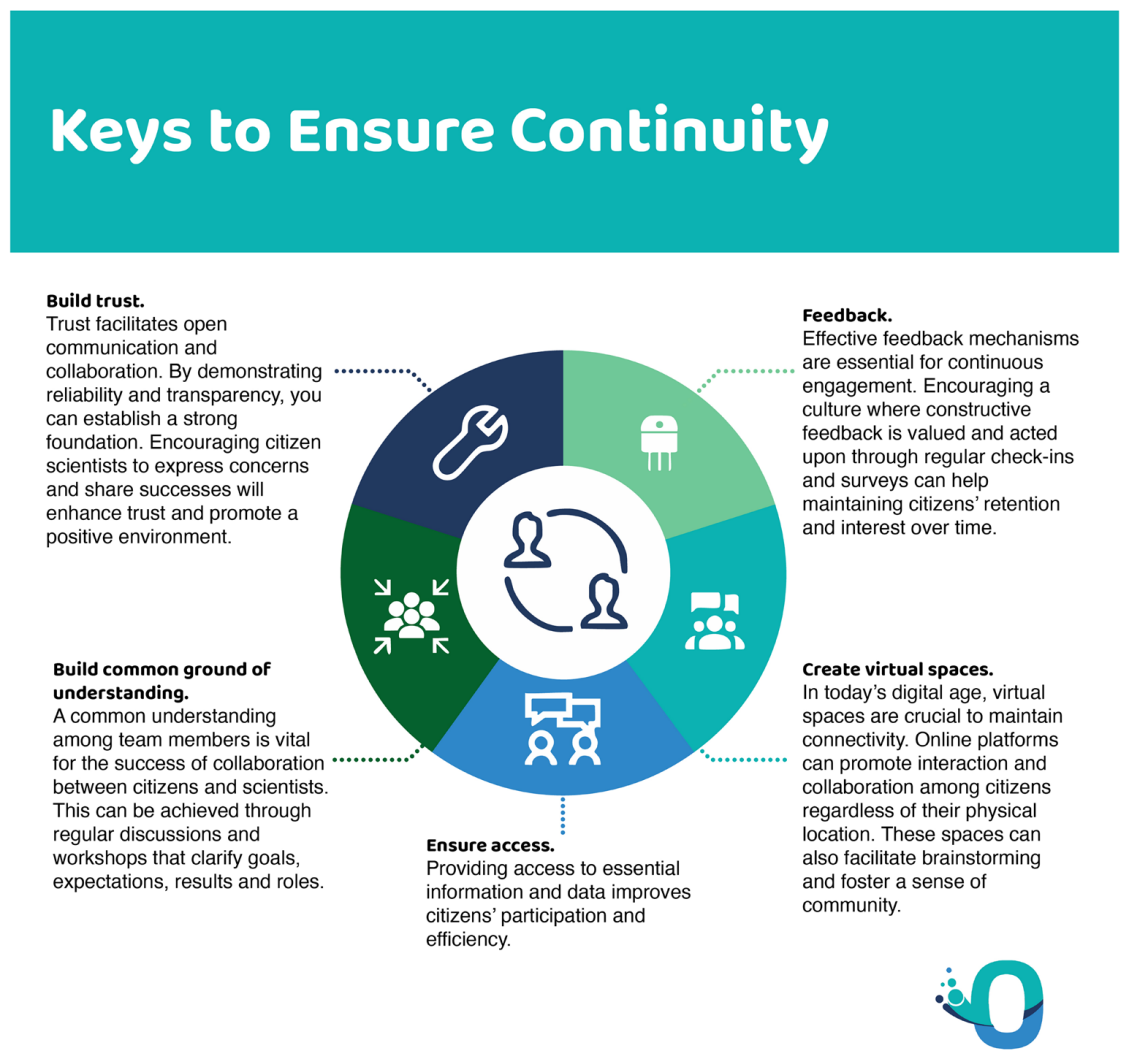
How to ensure continuity in citizen science.

What emerges from the literature is the need to establish an ongoing relationship between scientists, regulatory agencies and citizen scientists over time, building trust, shared understanding and access ( Figure 11). Understanding, for example of the scientific process, is often lacking among the general public but can be built up over time. Establishing trust also requires time and the development of a common language and vision. Access is fundamental to avoid a participant selection bias, a common challenge in citizen science. All three keys should be considered in the phase project design ( Altman *et al.*, 2023).

In terms of retention, the creation of virtual spaces that favour interactions between participants, as well as mechanisms for feedback based on their participation in the project can improve participant retention ( Torres *et al.*, 2023; Zhou *et al.*, 2020). Direct feedback from scientists is usually both a benefit to retention as well as an opportunity for knowledge exchange.

Projects that have the possibility to improve the ecological and chemical status of local watercourses are prime examples of opportunities to build relationships and a common shared vision. More efforts are needed from scientists and environmental agencies experts to help the creation of a network with the participants, by facilitating interactions and feedback in both directions.

### Citizen science and policy: opportunities for collaboration

What emerged from the publications consulted is the debate on the quality of the data acquired through citizen science. In the past many environmental agency experts - and scientists too - did not trust citizens enough in terms of quality control and measurement bias, where monitoring design and analytical methods were not considered sufficiently robust to be representative of the conditions of the water body. Another common observation in the literature is the idea that citizen scientist generated data is often underused for policy or regulatory purposes ( Carlson & Cohen, 2018; Woods *et al.*, 2022). This has been associated with the generally low trust in the limited quality control of citizen scientist generated data, which is also influenced by the often-limited diffusion of projects’ results as well as limited access to data, reports and publications. Incorporating citizen science insights into policy-making can lead to more informed decisions. Engaging citizens in monitoring environmental and societal changes ensures that policies are grounded in real-world data and community experiences, driving effective and sustainable solutions for the future. While projects can yield valuable insights, sharing these results is key to maximizing their impact. Effective dissemination (through infographics, reports, and interactive platforms) helps communicate findings clearly, inspiring new initiatives and allowing communities to act on the information provided from citizen science efforts. For this purpose, unlocking citizen science data for everyone is crucial. By ensuring open access to all data and correct metadata generated by citizen science projects, we enable researchers, policymakers, and the public to collaborate effectively. Citizen science data could be in fact a very valuable resource to monitor the progress towards the WFD and SDGs, since regulatory agencies are often limited to major water bodies and do not extend to remote areas ( Ballerini & Bergh, 2021; Bishop *et al.*, 2020). This transparency fosters engagement, a sense of ownership and responsibility, and trust in data.

### Citizen science and environmental agencies: addressing future challenges together

**Figure 12.**
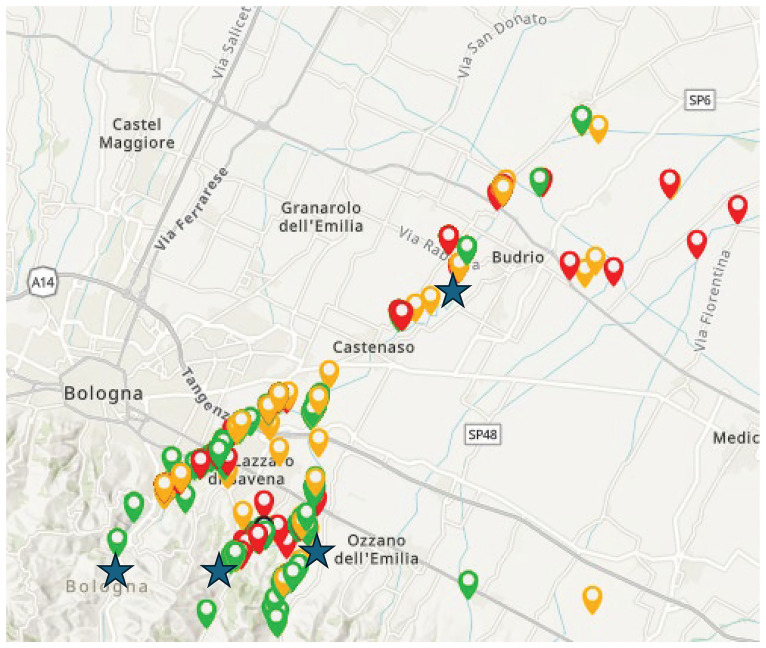
An example of virtuous collaboration between citizens and environmental agencies. Stars=official monitoring sites coloured points = citizens samples. This is an ongoing project where citizens are actively collaborating collecting data to support the regional environmental agency. This project is led by one of the co-authors of this article (Dr. Bruna Gumiero) and sees an active community of citizen scientists committed to freshwater monitoring. Data are available on the FreshWater Watch website, Idice project (Northern Italy).

Approximately 80% of watercourses are of first and second degree and therefore of small or very small dimensions, hard to cover by the official monitoring of national environmental agencies ( Kelly-Quinn *et al.*, 2022). Despite their size, small streams contribute to ecosystem services important for humans and biodiversity, including water regulation and erosion control. Additionally, those streams provide dispersal corridors along which aquatic organisms can move across the landscape and contribute to 80% of mean annual flow volume to bigger downstream reaches ( Ferreira *et al.*, 2023). Small streams are highly vulnerable to both local disturbances and geochemical characteristics of sediments. This means that point pollution of first and second order streams may enter the perennial drainage network and influence water quality causing diffuse pollution. The lack of governmental data on small streams can be attributed to the challenges of monitoring these water bodies. The extensive lengths and the complexity of the stream networks raise logistical difficulties including substantial time, labour effort and financial resources ( Kelly-Quinn *et al.*, 2022). As a result, small water bodies continue to be some of the least monitored freshwater resources, with notable gaps in spatial and temporal coverage. The distribution of official monitoring stations is often inadequate to identify point impacts along the main river channels. In recent years, several scholars have shown that citizen science can help fill the knowledge gaps left open by Environmental Agencies due to a lack of resources and time constraints ( Hadj-Hammou *et al.*, 2017; Haywood & Besley, 2014; Owen & Parker, 2018; Peeters *et al.*, 2023; Thornhill *et al.*, 2016; Wyeth, 2023). As long as Environmental Agencies have limited area coverage, citizen science then becomes a reliable tool to complement the monitoring of water bodies ( Figure 12).

However, the literature consulted highlights a general lack of a stable and diffused collaboration between citizen science practitioners and Environmental Agencies. Some virtuous examples exist, scattered across the world, as is the case of the UK, where the collaboration between governmental and non-governmental organisations has been historically strong, with projects like ARMI ( Brooks *et al.*, 2019; Moolna *et al.*, 2020; Thompson *et al.*, 2016), MoRPh ( England *et al.*, 2017), and PondNet ( Ewald *et al.*, 2018). Other examples can be found in the United States with RARE ( Kaufman *et al.*, 2017), and in other countries too ( Kim *et al.*, 2011; Venkatesh & Velkennedy, 2023). Some of these collaborations focus on the capacity of citizen science to produce timely data with the possibility of capturing exceptional events such as pollution incidents that may not be covered by traditional Environmental Agencies, prompting the intervention of the latter (ARMI). A less explored possibility offered by citizen science projects is to analyse the water quality of private water bodies such as ponds, which cannot be covered by Environmental Agencies (PondNet).

In recent years, a growing number of Environmental Agencies started using citizen science data to integrate their own databases ( de Sherbinin *et al.*, 2021; Thornhill *et al.*, 2016; Wyeth, 2023). To this end, a lot of work has been put into the formation and training of volunteers prior to monitoring and data gathering activities. The European Union has recognized the potential of citizen-generated data to inform the environmental policy landscape and to meet societal demands for more participatory decision-making. At the same time, the EU also identifies that contributions of citizen science to environmental policies are still limited, often due to the poor understanding of the benefits of citizen science. It is also important to determine whether projects that provide policy support also receive benefits to citizen engagement ( Turbé *et al.*, 2019). To this end, an overview of citizen science activities in support of environmental policies in Europe is provided in an inventory which is updated on a regular basis and with global coverage (Joint Research Centre Data Catalogue website).

## Examples of best practices

To assess the ecological status of rivers and streams according to the European WFD standards, freshwater monitoring examines three components: physicochemical state, biological communities and hydromorphology. These parameters can be effectively and efficiently addressed by citizen science projects. More recently, plastic pollution is a component being progressively more monitored in several marine and freshwater ecosystems across the globe with an increasing number of citizen science initiatives, suggesting this parameter should be included into the European WFD when specific monitoring standards are set and agreed upon on a European (and ideally, global) level, thus filling in monitoring and knowledge gaps.

### Chemical and optical monitoring

Most robust citizen science water quality projects are focused on optical conditions and basic chemical parameters, like pH, dissolved oxygen, turbidity, and nutrient concentrations. Of the latter, nitrogen and phosphorus-based measurements are usually the most straightforward allowing to detect eutrophication and nutrient pollution deriving from agricultural and livestock activities, industrial and wastewater treatment discharge, urban activities and runoff. Optical measurements, such as water colour using the Forel-Ule scale ( Malthus *et al.*, 2020) as well as Secchi tubes and disks for turbidity, have been used successfully in a wide range of citizen science projects on water quality ( George *et al.*, 2021). Basic chemical parameters such as pH and dissolved oxygen are sensitive to daily cycles, while low-cost measurements of specific conductance have proven to be particularly useful ( Shupe, 2017).

In this type of approach, the monitoring of water quality through citizen science is varied and widely implemented, covering watersheds as well as local scales ( Thornhill *et al.*, 2018). Studies show that properly trained volunteers can provide data comparable to those collected by professional scientists ( Fore *et al.*, 2001; Moshi *et al.*, 2023; Storey *et al.*, 2016). Coordinated citizen science initiatives can gather evidence on global freshwater ecosystems status, but despite this enormous potential to fill data gaps, world-wide efforts remain limited ( Jackson *et al.*, 2016). Most freshwater citizen science projects are place-based and therefore limited in geographic scope, resulting in a range of inconsistent efforts across the globe, with many projects working in isolation and with different analytical methods ( Cunha *et al.*, 2017; Walker *et al.*, 2021). However, some successful examples exist, like the **FreshWater Watch programme ( www.freshwaterwatch.org)**, created in 2012 by Earthwatch Europe as a globally consistent citizen science method to provide comparable data about the state of freshwater environments. FreshWater Watch core parameters are nitrates and phosphates, measured with an in-situ colorimetric assay, turbidity measured with the Secchi tube, and a series of standardized observations on the water body itself (like speed of water, presence of debris, algae, and general visible surfactants). Additionally, observations on the surrounding habitat are also recorded, like land use in the immediate proximity and type of vegetation present. The centralized, globally consistent protocol also allows to address and add local priorities on specific cases. The methodology and supporting website, mobile app., and training materials are translated into more than 20 languages and have supported over 120 projects in 25 countries, with nearly 15000 citizen scientists involved. Other programmes have followed, often focused on similar water quality challenges ( Blanco-Ramirez *et al.*, 2023).

### Biological communities

***Macroinvertebrates***

Macrobenthic communities exhibit dynamic responses to various environmental features, with a pivotal role played by pollutants. Pollutants were identified as significant contributors to adverse impacts on community well-being, affecting both taxa diversity and trait diversity. The evaluation of ecological quality relies on assessing the tolerance levels of different taxa to pollution, encompassing a spectrum of characteristics that respond differently. Consequently, lotic water environments impacted by pollution tend to exhibit more homogenous communities with minimal seasonal variability.

Beyond the direct impact of pollution, macroinvertebrates demonstrate correlations with other significant factors affecting freshwater ecosystems, such as morphological alterations that reduce riverine habitat diversity. Habitat availability, influenced by substrate nature and interstitial spaces, plays a crucial role in sustaining sensitive groups like Ephemeroptera, Plecoptera, and Trichoptera (EPT).

The management of riparian zones further influences the presence of sensitive taxa, with healthy riparian woods contributing to improved habitat quality, including cooler water temperatures, narrower channels, and enhanced food resource availability. Moreover, these areas serve as vital corridors facilitating the survival and dispersal of winged adults on land ( Greenwood *et al.*, 2012).

In addition to the environmental considerations, it is imperative to address the role of climate change. The anticipated increase in the frequency, severity, and duration of droughts poses a significant threat to macrobenthic communities. Even in the absence of noticeable chemical changes, alterations in hydrology exert a profound impact on macroinvertebrate assemblages. Water scarcity disrupts ecological processes such as microbial breakdown and the establishment of permanent periphytic biofilms.

Like chemical and optical tools employed by citizen scientists, variations in the assessment of macroinvertebrates are associated with different analytical methods. However, the variability is not only method-dependent but may also be influenced by the diverse target communities chosen for analysis. The research notes ongoing efforts to simplify official methods aligned with the WFD, despite variations in indices among member countries. The key determinants of this diversity include sampling methods, level of determination, and the reference list of target species. While the kick method and family level are commonly used, the list of target species exhibits considerable variability.

***Invasive species and fish communities***

In recent decades freshwater ecosystems witnessed a notable increase in invasive species, mainly attributed to globalisation and human activities, like intentional release. These invasions are currently one of the main threats to biodiversity, and their early detection is essential for a rapid and effective response for ecosystem preservation. Traditional knowledge of water bodies often relies on water practitioners like fishermen, and it is of uttermost importance as it can promptly address alert networks of alien species detection, swiftly transferring knowledge about detected changes to researchers and authorities. Recognizing the potential of local citizens, and especially fishermen, as early detectors of changes in river ecosystems and the introduction of exotic species becomes crucial in this context. Research indicates that citizen action at the ecosystem level can efficiently manage the presence of invasive species. Moreover, the development of citizen science programs not only has the potential to raise public interest in intervening against invasive species but also serves to keep citizens closely connected with scientific knowledge. A study by Clusa *et al.* (2018) conducted in Austria underscores the significance of incorporating citizens' knowledge and opinions in addressing biodiversity issues. Despite limited knowledge about invasive species, citizens demonstrated awareness of associated risks and exhibited a positive attitude toward eradicating these invasive species that affect native aquatic fauna, aligning with findings in a similar study conducted in Scotland. However, discrepancies surfaced between citizen-reported data and official reports concerning invasive species. Anglers, through techniques like electrofishing, accurately identified over 50% of native species, even detecting the presence of an exotic species, *Trachemys scripta*, a freshwater turtle that originates from North-east Mexico and South-east United States previously believed to be confined to artificial ponds. This emphasises the pivotal role of citizen scientists in supplementing official reports on invasive species. Another interesting result of the study conducted by Clusa *et al*. is that in water bodies of lower environmental quality, the knowledge about both native and exotic species was notably much more limited, potentially influenced by a perceived lack of aquatic fauna and the notion that conservation efforts were not worthwhile. Enhancing environmental education emerges as a potential solution to raise public awareness of invasive species and decrease their intentional release in aquatic environments. The combination of citizen science and molecular methods like environmental DNA (eDNA) presents a promising approach for early detection and monitoring of invasive species ( Larson *et al.*, 2020). Collaborative efforts, encompassing media, public education, and citizen science, are deemed crucial in preventing the spread of invasive species and fostering improved management programs for biodiversity conservation.

***Examples of citizen science projects and years of activity in biological communities monitoring***

**The Anglers’ Riverfly Monitoring Initiative (ARMI),** https://www.riverflies.org/riverfly-monitoring-initiative-rmi (active).

The Anglers’ Riverfly Monitoring Initiative (ARMI) is a citizen science project in the UK that empowers anglers and other river enthusiasts to monitor the health of rivers and streams by observing aquatic invertebrates, specifically riverfly populations. The initiative is a collaborative effort that brings together organizations like the Riverfly Partnership, Environment Agency, Scottish Environment Protection Agency (SEPA), Natural Resources Wales, and various conservation groups.

The project focuses on three key objectives: i) Monitoring River Health, as riverflies -such as mayflies, caddisflies, and stoneflies - are sensitive to changes in water quality so their presence or absence can indicate the health of a river ecosystem; ii) Early Warning System, the volunteers act as "first responders" detecting pollution events before they escalate into more significant issues; iii) Community Engagement, ARMI encourages community participation in conservation, creating a network of informed and proactive river stewards. The initiative is implemented through the following four steps:

1.   Volunteer Training: participants receive training to identify key riverfly groups and collect data systematically.

2.   Sampling: volunteers use kick-sampling techniques to collect riverfly specimens. This involves agitating the riverbed with their feet and collecting displaced invertebrates in a net.

3.   Data Collection: observations are recorded and shared with environmental agencies and local conservation groups.

4.   Trigger Levels: collected data is compared against predefined "trigger levels" for invertebrate populations. Significant deviations may indicate pollution or other ecological issues.

Like many citizen science projects, it offers numerous benefits, including enhanced surveillance that extends environmental monitoring beyond the capabilities of government agencies, faster detection and response to water pollution incidents, and increased public education and awareness, fostering a deeper understanding and appreciation of freshwater ecosystems.

Since its inception in 2007, ARMI has achieved remarkable success. Thousands of volunteers now monitor hundreds of sites across the UK. The initiative has played a crucial role in detecting pollution incidents, supporting water management efforts, and enhancing public appreciation of river ecology.

**RiuNet** https://www.fehm.cat/en/projectes-de-recerca-i-seguiment/ (University of Barcelona, Spain, active). RiuNet is a citizen science tool derived from the official IBMWP protocol (Iberian Biological Monitoring Working Party). A specific correlated mobile application was designed for smartphones and tablets, empowering citizens to evaluate the hydrological status and ecological quality of rivers in accordance with the guidelines of the EU Water Framework Directive ( Prat *et al.*, 2016). Using a simplified and interactive approach, this app guides the user to evaluate the ecological status with hydromorphological and biological quality tests. Utilising data derived from the official monitoring program conducted by the Catalan Water Agency (ACA), comparative analyses were conducted using a subset of RiuNet assessments within the inland basins of Catalonia. The findings underscore the invaluable contribution of citizens, revealing their capacity to provide pertinent information about the ecological state of rivers across the territory. Importantly, these contributions offer a more comprehensive understanding of river conditions, both spatially and temporally, compared to the limitations of official monitoring programs. Beyond its data-driven impact, RiuNet is driven by several overarching objectives. Firstly, it aims to draw attention to the ongoing degradation of rivers. Secondly, it seeks to enhance public awareness regarding the imperative need for the protection and restoration of these vital water bodies. Thirdly, the app strives to foster scientific engagement by encouraging the contribution of citizen-generated data. Lastly, RiuNet aims to elevate the level of understanding of river ecosystems, shedding light on lesser-recognized types, such as intermittent rivers or ephemeral streams, which are often overlooked in social discourse. Through these multifaceted goals, RiuNet emerges not only as a tool for ecological assessment but as a catalyst for positive change and public involvement in safeguarding our precious river ecosystems. RiuNet offered an interesting possibility for CS practice, as it requires a simpler sampling compared to the other methods, on the other hand it requires a good ability in taxonomic determination, as it comprehends 45 taxa mostly to be determined to the family level ( Verkaik *et al.*, 2019), which could pose a challenge for volunteers approaching freshwater macroinvertebrates for the first time.

**FLOW** https://www.flow-projekt.de/index.php/de/ (Germany, active). In the FLOW project, citizens learn how to assess and document the ecological condition of streams and small rivers in a standardised way. This standardised approach is imparted through comprehensive training courses and water surveys conducted across Germany. The primary objective is to establish an extensive database on the state of watercourses, contributing significantly to river research and laying the foundation for targeted protection and renaturalization efforts. Volunteers actively engage in collecting water body data, which is subsequently integrated into ecotoxicological and ecological studies. This collective effort forms the basis for developing local and regional water protection strategies. The FLOW program facilitates citizen scientists by providing training, support, and field equipment, empowering them to gather data on macroinvertebrate community composition, taxa abundance, and other key parameters. This includes calculating the SPEARpesticide bioindicator, assessing stream hydromorphology, and evaluating the physicochemical status of water bodies.

### Hydromorphology

The experience accumulated thus far in the implementation of the WFD underscores the imperative to assign greater significance to hydromorphology in ecological status assessment, monitoring, characterisation, and the formulation of effective measures. Specifically, there is a pressing need to substantially enhance the evaluation of hydromorphological pressures. It is crucial to recognise that hydromorphological processes manifest at diverse spatial and temporal scales, necessitating the utilisation of evaluation methods capable of accommodating these variations (multiscale methods). Although hydromorphology supports the diverse flora and fauna of our waters, and with the ever-increasing pressure of climate change and changes in politics, society and economics, restoring natural habitats in our aquatic environment is often not a priority. While the adoption of the hydromorphology concept has gained traction since the inception of the WFD, it is essential to note that it is currently relegated to a 'supporting element.' This means that, for water bodies where the ecological status falls below the “High” category, the hydromorphological state is not considered in the comprehensive assessment of the overall ecological condition. Furthermore, in Europe there are few methods sensitive to hydromorphological pressures, meaning that hydromorphological pressures and their effects can remain unnoticed in the assessment process. Consequently, this leaves member states to design programs of measures aimed at achieving good ecological status without a comprehensive understanding of all pertinent pressures and their impacts.

It is a common temptation in water management to hone in on singular issues, such as addressing point source pollution through enhanced wastewater treatment. Pressures and measures with easily identifiable sources often appear more manageable than hydromorphological pressures, which frequently stem from legacy issues or multiple stressors. The WFD underscores the principle of "cost-recovery" for water services, emphasising the need to appropriately "recover" financial, resource, and environmental costs from various water service users, guided by principles like the “polluter pays” principle.

In practice, implementing cost-recovery becomes a formidable challenge, especially in the realm of hydromorphology management. The intricacies of river restoration, for instance, often place the financial burden on the public purse, where budgets are constrained and public attitudes toward the imperative of restoring natural habitats can be variable. Moreover, public attitudes towards the necessity of restoring natural habitats introduce an additional variable into the equation. The perceived value of such endeavours may fluctuate, making it difficult to garner consistent public support for the financial investments required. Thus, achieving the ideal of cost-recovery in hydromorphological management proves to be a multifaceted challenge, requiring not only financial ingenuity but also a nuanced understanding of public perceptions and a commitment to navigating the complexities inherent in ecological restoration. Citizen science activities can be highly valuable for monitoring and appraising physical habitat changes in rivers. Local volunteers often have firsthand knowledge of their environment, allowing them to quickly notice and report physical changes, such as sediment deposition, erosion, or alterations due to extreme weather events. Citizen scientists also have historical knowledge of river flow patterns, vegetation, and land use. As participants monitor physical habitat changes, their environmental literacy increases on issues like pollution, habitat degradation, and invasive species, leading to advocacy for river conservation.

***Examples of hydromorphology citizen science projects and years of activity***

**The Modular River Survey (MoRPh)** https://modularriversurvey.org/morph-rivers/ (UK, active). MoRPh is a valuable tool for citizen scientists engaged in monitoring river channels and riparian physical habitats. Developed in 2016, MoRPh has gained gradual adoption across England, Wales, and the Republic of Ireland. This survey methodology allows citizen scientists to collect data at different spatial scales, providing insights into river morphology and functioning. Key features of MoRPh include its ability to complement biological surveys by characterising the physical structure of river channels and vegetation. Unlike many citizen science surveys that focus on biological or water quality aspects, MoRPh fills a crucial gap by addressing the physical structure of rivers. The survey tool, accessible at https://modularriversurvey.org/, offers manuals, field guides, survey forms, and indicator formulations for public use. The MoRPh survey methodology covers physical habitat, vegetation structure, sediments, geomorphic features, and human interventions and pressures. Geometric/visual guidance is provided to aid non-specialist citizen scientists in data collection. After uploading surveys to the web-based information system, values for 14 indicators are calculated and mapped. The indicators synthesise natural properties and assess anthropogenic influences. Over 350 active citizen surveyors have conducted approximately 2300 MoRPh modules, revealing variability in indicator scores across surveyed lowland rivers in England and Wales, with potential for future surveys in upland rivers.

**The citizen River Habitat Survey (cRHS),** https://www.therrc.co.uk/crhs, (UK, active). The River Habitat Survey (RHS) is an established standard methodology for characterising and assessing the physical character of freshwater streams and small rivers. The methodology is used across the UK and has a database of >25,000 sites, with 2,500 in Wales collected since 1994. RHS data is used to calculate a series of quality scores relating to the hydromorphological condition of rivers that can support WFD assessment including habitat modification score, habitat quality scores, riparian quality indexes and river habitat quality index. The data is widely used in the environmental sector to support planning, management and river restoration and it was applied towards assessing the state of the environment in Wales, the implementation of the Water Framework Directive and prioritising for river restoration. The River Restoration Centre (RRC) has adapted the RHS for citizen science (cRHS) so that it can be applied by members of the public after attending a short training course. The data will be used to introduce citizen scientists to hydromorphology, the science describing the way rivers shape and maintain habitats for species. The aim of cRHS is to have citizen scientists collect, input and interpret habitat data with the help of more experienced surveyors so as to produce assessments of habitat quality and river restoration plans and projects. The cRHS involves recording habitat features, engineered structures and other pressures and taking measurements, photos and videos.

**Catchment Based Approach,** https://catchmentbasedapproach.org/learn/, (UK, active). The Catchment-based Approach ( Catchment Based Approach, CaBA, 2023) is a water management initiative that promotes the collaborative working of volunteers, governmental agencies, farmers and business companies at a river catchment scale. Established by the UK Government Department for Environment in 2013, CaBA has operated in England and Wales. Its partnerships are currently active in more than 100 river catchments, engaging over 43,000 primary stakeholders. The CaBAs projects concern a range of themes from rural land and urban water management to habitat restoration, natural flood risk management and water quality monitoring. Among those themes, habitat restoration activities involve the mitigation and removal of barriers to fish migration or the identification and eradication of invasive vegetation. Stakeholders and volunteers involved in CaBAs projects are trained and supported by experienced scientists in a wide range of technical areas, including the application of modelling tools using Geographical Information Systems (GIS) ( Collins *et al.*, 2020). Further, training sessions through webinars and technical guides are available in the CaBa website ( https://catchmentbasedapproach.org/learn/).

### Riverine and freshwater plastics

Citizen science can contribute in collecting riverine plastic data, as, for other parameters, it can provide high temporal and spatial scales at reduced costs with respect to the professional monitoring by research institutes and environmental agencies. Additionally, the fact that the public is engaged in the research process facilitates the awareness of the plastic problem and its sources, promoting the creation of zero-pollution attitudes and a behaviour towards litter reduction at its source ( Popa *et al.*, 2022). Available data collected by citizen scientists on riverine litter presence can include floating macrolitter, riverbanks litter, and microplastics. However, in general citizen science projects targeting riverine plastics are mostly focused on macrolitter as the visual identification and characterization of larger debris does not require specific tools or laboratory equipment otherwise not accessible to citizens. In some projects citizens were engaged in the collection of water samples, but the analysis to identify microplastics (< 5 mm in size and smaller) was conducted by research staff in laboratories equipped with microscopes and other instrumentation, so citizens were not involved in the analysis or interpretation of the data (e.g. Barrows *et al.*, 2018; Kiessling *et al.*, 2023 and Kiessling *et al.*, 2021). The projects’ approaches can vary, and can include co-design and data interpretation, as well as via personal initiatives or crowd-based observations where the direct interaction with the volunteers is absent, participants are asked to follow certain guidelines, and either ship samples to a laboratory or upload photos and observations of litter and plastics through a custom-developed app for smartphone. In the latter cases, the area coverage is wider, reaching otherwise inaccessible places, and data collection is relatively quick and cost-effective. The temporal and spatial coverage may ultimately counterbalance less precise individual measurements and the lack of data validation ( Van Emmerik *et al.*, 2020), but citizen engagement and the project’s reach might be limited to already environmentally active communities.

When the citizens receive enough training and clear guidelines, and there are criteria in place for data validation, citizen scientists’ data are reliable as those of professionals: in fact, missing information rather than methodological errors are the limiting factor in many citizen science projects ( Kiessling *et al.*, 2021). Some of the projects summarised below deal with both microplastics and macroplastics, but have different monitoring and engagement strategies, as well as different target volunteers (schools versus communities or single citizens). For the initiatives identified in this work there are a very few projects on riverine plastics that could encompass all characteristics of citizen science: co-design, social interaction, data validation, samples analysis and data interpretation by citizens and volunteers. Most projects are either “tools” useful for citizen science where there is no interaction with the citizen (e.g. apps for counting floating litter, which however are repetitive, are not harmonised in terms of plastic litter categories for classification, and could just be unified into one global initiative or into the same guidelines to allow data intercomparison); tools that rely on already engaged communities; or the projects do not actively involve citizens in the analysis of the samples and data interpretation (samples are shipped to a laboratory). This latter is particularly true for projects aiming at analysing microplastics. Harmonisation of monitoring efforts (floating and riverbanks litter survey guidelines for example), classification guidelines (plastic and other litter categories), are urgently needed. A citizen science microplastics cut-off size should be decided (e.g. all plastics visible to the naked eye, > 1 mm) allowing for the exact determination of the materials of particles collected as plastic/not plastic (e.g. by the melting approach) after classification according to size, colour, shape, etc. Colour classification for both macro and microplastics should follow a clear protocol and colour codes (e.g. Pantone colours system, or the open-source RGB colour codes). Timing of surveys (for floating litter as well as for nets for microplastics) should be harmonised. The size and shape characteristics of areas of river/lakes banks surveyed for stranded litter should be unified (e.g. transects, circles, squares, and how extended these areas should be), and replicates and blank controls should be decided. Ultimately, in rivers and estuaries, the use of remote-sensing devices like cameras and drones by trained groups of citizens could help the detection of plastic debris allowing the identification of point sources of leakage and a prompt intervention. All these implementations could help make citizen collected riverine plastic data comparable, robust and useful in research studies as well as in informing policy making strategies. Useful guidelines on the visual identification of plastic particles were just published ( Markley *et al.*, 2024) as a first step for providing ease of access and affordability to microplastic identification for broad use across volunteer groups, research labs, organisations, and others.

***Examples of citizen science projects and years of activity in monitoring riverine and freshwater plastic***

**Plastic Pirates and Plastic Pirates Go Europe!EU, https://www.plastic-pirates.eu/en** (Germany and Europe, 2016–2024). Plastic Pirates is a citizen science campaign which contributed to the research on the distribution of macro- and microplastics along German rivers and riverbanks. It was part of the Science Year 2016/17 - Seas and Oceans and of the research focus “Plastics in the Environment” of the German Federal Ministry of Education and Research (BMBF), and carried out by the Ecologic Institute in cooperation with ozean:labor at Kieler Forschungswerkstatt, 2016 – 2020. In the project, school classes and youth groups collect plastic samples from streams and rivers and document their findings. The collected data is then analysed by scientists and researchers, making an important contribution to researching the state of European rivers and the extent of pollution caused by plastic waste. The project has been upscaled by PlasticPiratesEU (Horizon Europe project, https://www.plastic-pirates.eu/en) and the Plastic Pirates – Go Europe! initiative launched by the Trio-Presidency of Germany, Portugal and Slovenia into a pan-European citizen science initiative (2020). In Plastic Pirates, approximately 5500 schoolchildren participated in the sampling, forming 408 project groups from about 340 schools and youth organisations throughout Germany, sampling from small rivers and channels to major rivers ( Kiessling *et al.*, 2021). Participants were provided with a guidebook with sampling instructions and a booklet with background information about environmental litter pollution for local supervisors. Samples were taken for floating macrolitter (> 25 mm), meso (24.99 - 5 mm) and microplastics (1–5mm) ( Kiessling *et al.*, 2021), and for riparian litter ( Kiessling *et al.*, 2023). Floating litter is visually observed and counted in at least 30 minutes surveys, taking pictures whenever possible; meso-and-microplastics are samples with custom-made nets with a mesh size of 1000µm, deployed for 60 minutes. Participants are also asked to quantify the water velocity in three repeated measures by throwing a wooden stick in the water and recording the time it needs to pass from two points 20 m distant from each other along the riverbank. Riverside litter is instead recorded in an area of at least 1000 m ^2^ by establishing up to three transects perpendicular to the river course, each transect with a defined number of sampling stations, in predefined zones: the river edge (0–5m distance to river, regular contact with the river water), the river bank (5–15m distance to river, irregular contact with water of river during flood events), and the river crest (15m or more distance to river, not in contact with the river) ( Kiessling *et al.*, 2023). The litter items are counted by participants in circles with a radius of 1.5 m and classified according to the following categories: paper, cigarettes, plastic, metal, glass, food leftovers, and other items. The datasets collected are stepwise validated with the schools mentors and supervisors and only complete datasets that report correct measurement times and a proper identification of the sampling site are considered. Samples collected are sent to the laboratory of the Kieler Forschungwerkstatt for polymer identification.

**The Global Microplastics Initiative and the Gallatin Microplastic Initiative https://www.adventurescientists.org/microplastics.html**, (Global and US, 2013 – 2017) The project aimed at better understanding microplastics concentration and types in marine and freshwater ecosystems across the globe. The project involved several worldwide volunteers between 2013 and 2017. Volunteers received a field protocol based on the use of a 1-L sampling bottle and on the “grab method” adapted from EPA sampling protocols. Participants belonged to outdoor communities (hiking, mountain biking, kayaking, mountaineering), and ambassador professional athletes recruited volunteers through their networks. Once in the project, volunteers were required to complete an online training and pass a test. Participants collected samples in locations they frequently or occasionally visited. The project yielded 2,667 samples for the Global Microplastics Initiative from all over the world. Samples were shipped by the participants directly to the Ocean Analytics in Deer Isle, Maine, for microscopy analysis of the particles in their 1-L bottles. A subset of samples randomly selected underwent µFTIR (Fourier-transform infrared spectroscopy) analysis. The dataset is composed of 66% marine samples and 34% freshwater samples. By applying the same protocol for freshwater environments, Adventure Scientists implemented the Gallatin Microplastic Initiative ( Barrows *et al.*, 2018), which had the aim to examine the presence, size, and type of microplastics in the Gallatin Watershed over two years and to describe the seasonality of microplastic pollution in the headwaters of a watershed. In the Gallatin River Watershed 72 sample sites were selected for seasonal collection. Of these, 22 sites were along the mainstem of the Gallatin River and 50 sites were from tributaries. The method used for sample collection was still the “grab” method and in this initiative, as opposed to the Global effort, pre-trained volunteers visited pre-assigned sample sites four times per year (September, December, March, and June, according to the hydrological flows), in a sampling window of 10 days. A total of 774 samples were analysed and other parameters collected included temperature, coordinates, and site substrate type. Samples from the Gallatin Microplastic Initiative were also shipped to Ocean Analytics. The results were shared with volunteers and at the end of the projects all participants (both global volunteers and Gallatin River volunteers) were asked to complete a survey that assessed volunteer experience, project impact and conservation outcomes.

**POSEIDOMM** www.poseidomm.eu, https://cordis.europa.eu/project/id/702747 (Italy, 2016–2018). POSEIDOMM was an EU-funded project within the Marie Skłodowska-Curie Actions focused on microplastics, that had an important aspect of citizen engagement through citizen science in the Arno river watershed in Central-Northern Italy. The citizen science initiative of the POSEIDOMM project consisted in the engagement of about 40 volunteers between 2016 and 2018, including school kids with their teachers and retired people, in the seasonal (5 times per year) monitoring of chosen sampling sites along the Arno river, its tributaries and a couple of lakes for water quality parameters (nutrients, turbidity, riverbanks conditions, presence of algal blooms) through the FreshwaterWatch platform, which had been modified to include banks’ macrolitter. In the POSEIDOMM project citizen science initiative, banks’ litter was collected in an area of 20 x 20 m along the rivers’ or lake’s shore, catalogued, and properly disposed afterwards, following a similar initiative ( Levesque *et al*., 2017). The categories for classification of the litter items found were based on the activity originating them: a) shoreline and recreational activities (bags, beverage bottles/cans, 6-pack holders); b) fishing activities (bait containers, fishing lines, fishing lures); c) smoking-related activities (cigarettes/cigarette filters, lighters, tobacco wrappers); d) dumping activities (appliances, car parts, tires, building material); e) medical/personal hygiene (condoms, diapers, syringes, tampons/tampon applicators); f) other debris/items of local concern (discarded food, firework debris, drug). The number of items belonging to each litter category was estimated using four abundance classes (0, 1, 2–10, >10 items). In the 2 years project, over 1000 macrolitter items were collected and removed from rivers and lakes’ shores, results that contributed to inform the local administration about point sources of litter and dumping sites. Prior to data collection, group citizens’ training and multiple joint monitoring with the researchers were performed. Data were validated with the researchers as soon as the data were uploaded on the FreshwaterWatch subproject’s web space, accessible by all project’s participants, and in-person in several informal meetings to discuss the results. When doubts arose in the group of citizens and schoolkids for any water quality parameter data and riverbank litter, a constant communication channel was in place with the researchers to discuss any issue.

**TrashAI** https://www.trashai.org/ (Global, active). TrashAI is an open-source code that can be used by any citizen who uploads images of litter from any environment. It can be a powerful tool for the classification of litter and to get back data about the trash in the image, including the classification of trash and the bounding box of where the trash is in the image. Data validation is made with AI and the open-source code can leverage citizen science collected data on riverine plastic pollution provided that enough information is given on the sampling sites where pictures are taken. In this project, anyone can contribute but the social aspects of citizen science are not implemented. The code can be seen as a very useful service and implementation for citizen science projects on any type of habitat.

**The Ocean Cleanup** https://theoceancleanup.com/research/citizen-science/ (survey app for rivers, global, active). The Ocean Cleanup has implemented an app (The Ocean Cleanup Survey App for Rivers) that allows tracking plastic debris transport in rivers. Citizens are required to find a safe location (a bridge is ideal) over their nearest or chosen canal, stream or river, and start counting objects that float by, by using this app on the smartphone or tablet. The data are used by the Ocean Cleanup to refine global river transport models and to identify pollution hotspots where to concentrate cleanup efforts. The app provides a short guide for monitoring including information on the best position for counting floating litter. The categories under which floating plastic objects and other debris need to be classified are: a) hard plastic (crates, baskets, toys..); b) soft plastic (plastic bags and wrappers); c) foam (Styrofoam disposable items); d) bottles of any kind; e) other plastics (diapers, nappies, sanitary products); f) clothing & textiles (shoes, garment, nets, strings, clothes, textile bags); g) organic (wood, seaweed, leaves); and h) human non-plastic (metal, glass, paper, cardboard, rubber). The location of the survey is shared by GPS through the tablet or smartphone. However, there is no information on if and how the data are validated since volunteers are only required to report the number of floating items per category they identify. Likewise, the social component of citizen science is not implemented in this project, including citizens training, so the app can rather prove to be a useful tool to complement other more structured citizen science initiatives focused on riverine and freshwater habitats.

**Preventing Plastic Pollution** by The RiversTrust (UK, active). https://preventingplasticpollution.com/about-the-project/ https://theriverstrust.org/our-work/our-projects/preventing-plastic-pollution-ppp Rivers Trusts in the United Kingdom aims to protect and restore freshwater ecosystems. This is done also through citizen science opportunities that encompass water quality monitoring, assessment of polluting outfalls, surveying riverine plastic pollution, mapping and control of freshwater invasive species and assessment of the biological health of rivers ( Collins *et al.*, 2023). Rivers Trusts worked in partnership with 18 organisations from across France and England in a project called “Preventing Plastic Pollution”, sought to understand and reduce the impacts of plastic pollution in the marine and freshwater environment. By looking at the catchment from source to sea, the project identified and targeted hotspots for plastic, embedded behaviour change in local communities and businesses and implemented effective solutions and alternatives. The project created an initiative to pick and monitor litter from source to sea to address the lack of data in river catchments and amplify the efforts of existing litter picking groups. Volunteers were trained to use standardised survey methods, aligned to the OSPAR Commission’s guidelines for monitoring marine litter ( Wenneker *et al.*, 2010) to ensure data comparability, while Rivers Trusts created and open access data platform on plastics where all guidelines and resources, survey findings, data visualisation and export were accessible by the community groups. The platform had the objective to provide information on similar surveys across the UK to provide users a comprehensive magnitude of the issue and of similar initiatives, and allowing volunteers to seek and join new groups and initiatives, thus helping in engagement and recruitment. The Preventing Plastic Pollution project was approved by the Interreg France (Channel) England Programme, and worked across seven pilot sites: Brest Harbour, Bay of Douarnenez, Bay of Veys, Poole Harbour, and the Medway, Tamar, and Great Ouse estuaries.

**CrowdWater** https://crowdwater.ch/en/welcome-to-crowdwater (Switzerland and Global, active). CrowdWater is a SNF-funded project at the University of Zurich, Department of Geography, Unit Hydrology & Climate. The long-term goal of the project is to collect a large number of observations and thus improve the prediction of hydrological events such as drought or flooding. To reach these goals, the CrowdWater app is used to collect data in various categories:

•   Water level data with physical and virtual staff gauges

•   Qualitative data on soil moisture

•   Data on the dynamics of temporary streams

•   Data on the documentation of plastic pollution in and around water bodies

•   General data on various watercourses

All data collected is published in the data overview. Current research focuses of the project are the use of data on temporary streams and the implementation of the citizen science approach to collect water quality data. Users can download the app and record floating macroplastic items, or stranded plastic items on the rivers’ shore ( Van Emmerik *et al.*, 2020). The location of the monitoring is provided with the GPS. No information on co-design, citizens training, data quality and validation is provided, and as for the Ocean Cleanup app, this project relies on citizens’ observations, constituting a useful tool to complement more structured citizen science approaches.

**Pescadores de Plastic (Spain, 2019–2023)** https://mon.uvic.cat/pescadors-de-plastic/). The project aimed at assessing the presence of plastic pollution in Catalan rivers and investigating the role of these systems as transporters of plastic waste from terrestrial to marine ecosystems through citizen science carried out by school children. One of the objectives was also to promote scientific culture among school children, to increase citizen engagement in scientific monitoring, and to upraise the public awareness of the impact of plastic pollution in aquatic ecosystems and the role of rivers in litter distribution. The project saw school kids as citizen scientists in the co-design of research questions, research steps, and analysing, interpreting, discussing and communicating results under the supervision and support of the researchers. Groups of school kids chose a river that was monitored following sampling guidelines and with the help of a sampling kit. Pre-monitoring workshops provided information on the standardised methods for sample processing of macro- and microplastics, and the results were discussed and validated together with the researchers’ coordination team. The project’s protocol is based on the guidelines for macrolitter surveys in rivers of the Chilean project “Cientificos de la Basura” ( http://www.cientificosdelabasura.cl/). In 2023, the Pescadors de Plastic project adhered to Plastic Pirates continuing the activities through the Plastic Pirates network and the Plastic Pirates Go Europe initiative, by using the project’s sampling protocols and approaches.

**Community-Driven Freshwater Plastic Monitoring in Western Africa** https://www.museumfuernaturkunde.berlin/en/science/community-driven-freshwater-plastic-monitoring-western-africa (Western Africa, March - October - 2023). This project is funded by UNEP and follows the UNEP guidelines for plastic monitoring in rivers and lakes ( UNEP, 2020). The project addresses plastic pollution in the Odaw river basin in the Greater Accra Region of Ghana. The Republic of Ghana is facing a plastic pollution crisis due to inefficient recycling and mismanaged plastic waste. This results in high plastic loads on land and in aquatic ecosystems, posing serious health concerns as well as socio-economic repercussions on the population. This project stems from a collaboration between the Museum für Naturkunde Berlin (MfN, Germany), Wageningen University & Research (WUR, Netherlands), and the Helmholtz Centre for Environmental Research (UFZ, Germany). The project’s aim is to understand the role played by citizen science in this monitoring effort, and its effects on the community involved. At first, citizens are engaged in plastic pollution monitoring according to UNEP guidelines; subsequently, their level of knowledge, awareness and further engagement in environmental plastic pollution issues is assessed based on their recent citizen science activities. The government therefore opened a platform for solutions on reducing plastic waste, that includes various stakeholders and addresses the role of citizen science in plastic monitoring, activity to which the project contributes.

### Navigating the way forward

From the projects and publications analysed in this paper, it emerged that scientists embarking in citizen science projects do not only aim at scientific advancements and a growing knowledge of the surrounding environment, but also to a sensible impact on participants, especially in terms of attitudes’ change and increase of scientific knowledge and scientific literacy. How to achieve these objectives is yet to be fully determined, but some good practices have been developed to reach objectives such as proper citizen involvement and sustained efforts in time. Addressing the social impacts of citizen science projects on participants is a promising and expanding field of study, outlined in some key aspects below.

**Emotional engagement can be a lever of change** to solicit environmental consciousness, and increase participation and retention over time. For example, across the globe, a growing number of rivers is being recognized as a living entity and pushed by the rights-of-nature movements, rivers are being given legal personhood or the right to flourish and be safe from pollution. This is the case of the Magpie River in Quebec, Canada, but other examples exist in Australia, New Zealand and Bangladesh, where local authorities have acted to protect rivers by granting them legal entities. In Ecuador the rights of nature are enshrined in the constitution since 2008, while Bolivia, Mexico and Colombia have created similar legal mechanisms to protect nature. In the United States, residents of Toledo formulated a bill of rights for Lake Erie. The Universal Declaration of Rivers’ Rights (UDRR, https://www.rightsofrivers.org/) encompasses some of the fundamental values that could be attributed to rivers and proposes six minimum rivers’ rights: (1) right to flow, (2) to perform essential functions within its ecosystem, (3) to be free from pollution, (4) to feed and be fed by sustainable aquifers, (5) to native biodiversity, and (6) to regeneration and restoration (UDRR, https://www.rightsofrivers.org/). The UDRR also suggests that these rights are possessed by the whole river basin, calling for guardians to act on behalf of river rights. Several governments have referred to UDRR for their legislation promoting river rights, such as El Salvador (Lempa river), France (Tavignanu river), Mexico (Oaxaca rivers), Nigeria (Ethiope river), Pakistan (Indus river), UK (Frome river), and Serbia. On a smaller scale, the study of small streams and small water bodies, especially those of private property and often lacking a proper monitoring (if any at all) by environmental agencies, can contribute creating a sense of belonginess in participants.

**Environmental citizenship,** defined as a citizenship guided by green ideas that results in environmentally friendly attitudes and actions, highlighted as need to promote the transition to sustainability ( Barry, 2002; Bauer *et al.*, 2020; Dobson & Bell, 2006) is an interesting approach in the landscape of behaviours and initiatives to respect and foster nature’s rights. Experts have identified strategies to encourage environmental citizenship within citizen science projects and help change attitudes towards the environment: “First, collectiveness: a key component of citizenship is participation in the collective. Second, situatedness: citizen science initiatives need to cultivate situated citizenship. Third, connectedness: citizen science projects should help their participants make connections between the data they collect and larger environmental problems” ( Jørgensen & Jørgensen, 2021), while others have pointed at the fact that scientists need to approach their projects from different social dimensions to encourage social learning and attitudes’ changes ( Fielke *et al.*, 2022). Social learning, or collaborative learning, is here defined as the process of specific knowledge sharing that happens in a structured or unstructured way between stakeholders and scientists, thanks to which both can learn something more about the problem at hand ( Mackenzie *et al.*, 2012).

**Environmental learning and education** help integrating into citizen science projects not only those citizens already environmentally conscious and receptive, but schools, students and teachers in particular. Citizen science holds considerable potential for science education and learning ( Bonney *et al.*, 2009). Schools’ engagement can be challenging but worth pursuing (Gumiero, EU project MICS grant nr. 824711 and Galgani, EU project POSEIDOMM, Grant nr. 702747, personal communication). Citizen science can support two types of education: formal and informal learning. Formal learning refers to lessons plans based in existing learning environments such as schools and universities, co-designed by scientists and teachers where students become willingly or not volunteers of the project ( Roche *et al.*, 2020). Informal learning is to be appreciated in non-traditional learning contexts, when participants find themselves learning science or the natural world surrounding them even though no formal learning plan had been established in advance by the researchers. The power of citizen science projects lies naturally in the informal learning, as it provides a valid alternative in the learning process of hard sciences, subjects that students tend to consider more challenging ( Araujo *et al.*, 2022). There are some major advantages in involving schools: first and foremost involving schools means opening them to actual science and research, possibly creating networks of schools and researchers and connecting different schools too. Another benefit is the widening of the engagement, as including more participants beyond already perceptive individuals is an impactful goal. Not to forget are the spillover effects when students talk with their families in terms of scientific knowledge, as well as what teachers have to offer, which goes from dissemination within their classes to more willingness to participate in future projects, all aspects supporting long-term engagement.

To address the challenges faced by aquatic environments, a structured European framework (the European Union Mission “Restore our Ocean and Waters by 2030”) was launched in September 2021 with the aims to “protect and restore the health of our ocean and waters through research and innovation, citizen engagement and blue investments, addressing the ocean and waters as one, playing a key role in achieving climate neutrality and restoring nature” (EU Mission “Ocean and Waters” website). To reach this objective, broad public mobilisation and engagement are cross-cutting actions that the EU identifies as relevant levers of change. When emotional engagement, environmental citizenship and education in citizen science projects have several social benefits on the participants fostering behavioral change, citizen-generated data need to be accurate to provide a meaningful scientific impact in support of environmental policy.

In citizen science, to reach data accuracy (as the “degree to which data are correct overall”, Kosmala *et al.*, 2016), project processes should be designed and structured with a clear end in mind to gain quality and useful data, according to the principle of *fitness for use* ( Bowser *et al.*, 2020). This concept highlights the fact that data should meet specific requirements of its intended application, because accuracy and precision in a generic sense are not sufficient. Data might be technically accurate but unfit for a particular purpose when collected in inconsistent ways or lacking resolution, coverage or metadata. Since data standards in citizen science are still lacking, it is difficult to assess its *fitness for use* by professionals, researchers, or policymakers. Without a clear and explicit appropriateness for application, citizen-generated data can unfortunately be dismissed, losing an opportunity to gather information and answer important question.

To meet the specific project research objectives, citizen science needs norms, guidelines, and standards. In the assessment of chemical and ecological status of freshwater environments, data standardization in citizen science allows a clear understanding of its strengths and limitations facilitating its integration into formal research and decision-making. To gain trust and a proper use of citizen science data, metadata, documentation of methods, and quality assurance are essential as it is the case for professional scientific approaches. Citizen science data should also align to professionally gathered data across diverse environmental conditions, and for a minimum project lifetime based on the peculiarity of the ecosystems being studied (e.g. the variety of anthropic impacts or the proximity to point-sources and nonpoint sources of pollution, recurrence of extreme events, or any noticeable change).

Ultimately, it is strongly suggested to cross-validate citizen science data at the start of each new project and methodology by pairing it to the monitoring activity to professional scientists. This initial cross-validation might serve to set the basis for standardised monitoring guidelines for different ecosystems (rivers, streams, lakes, ponds, etc..), habitats, ecological indicators (macroinvertebrates, fish communities, keystone species) and pollutants (nutrient concentration, plastics). The criteria of data accuracy, standardization and validation underscore the commitment to the reliability and utility of citizen-generated data in advancing our understanding of aquatic ecosystems and guiding effective conservation efforts.

## Data Availability

The underlying data for the analysis on the publications addressed for this paper is openly available in Zenodo ( https://doi.org/10.5281/zenodo.14633986). Zenodo: Publications Database for Freshwater Citizen Science Projects to address WFD and SDGs objectives. https://doi.org/10.5281/zenodo.14633986. ( Galgani *et al.*, 2025) The project contains the following data: WP1_ORE_dataZenodo.xlsx
