# Supplementary material for: Freshwater monitoring across the globe: the role of citizen science within the European Water Framework Directive (WFD) and the United Nations Sustainable Development Goals (SDGs), and opportunities to incentivize the collaboration with environmental regulators
Source: Open Res Eur. 2025 Aug 20;5:45. Originally published 2025 Feb 7. [Version 3] doi: 10.12688/openreseurope.19162.3 (PMC12405854; doi:10.12688/openreseurope.19162.3)
Supplement: Peer review history

# Reviewer report: Reviewer response for version 3

**DOI:** 10.21956/openreseurope.22903.r58723

**Published:** 2025-09-02

**Authors:** Rajaram Prajapati

**Affiliations:** 1 University of Pittsburgh, Pittsburgh, PA, USA

The authors revised the manuscript based on my comments. I have no more comments.

Where applicable, are recommendations and next steps explained clearly for others to follow? (Please consider whether others in the research community would be able to implement guidelines or recommendations and/or constructively engage in the debate)

Partly

Does the article adequately reference differing views and opinions?

Partly

Are all factual statements correct, and are statements and arguments made adequately supported by citations?

Yes

Is the rationale for the Open Letter provided in sufficient detail? (Please consider whether existing challenges in the field are outlined clearly and whether the purpose of the letter is explained)

Partly

Is the Open Letter written in accessible language? (Please consider whether all subject-specific terms, concepts and abbreviations are explained)

Yes

Reviewer Expertise:

Citizen Science, Hydrology, Water Monitoring

I confirm that I have read this submission and believe that I have an appropriate level of expertise to confirm that it is of an acceptable scientific standard.

# Reviewer report: Reviewer response for version 2

**DOI:** 10.21956/openreseurope.21644.r54843

**Published:** 2025-06-14

**Authors:** Rajaram Prajapati

**Affiliations:** 1 University of Pittsburgh, Pittsburgh, PA, USA

General comments

1. The title uses an unexplained abbreviation—consider spelling it out for clarity.
2. This review tackles an important topic and brings together a broad set of examples on citizen science in freshwater monitoring. While the effort is commendable, the paper would benefit from clearer structure, less repetition, and a more transparent methodology. Key points—like participant engagement, data quality, and collaboration with agencies—are relevant but could be discussed with more critical insight. With some tightening and clearer framing, it has potential to make a valuable contribution.
3. illustrating key results or methods. I recommend keeping only those that directly support the paper’s arguments, ensuring each is cited in the text and accompanied by a more descriptive caption.

Introduction

1. Currently, the paragraphs read like standalone blocks and lack logical connection.
2. While it’s important to situate the paper in the context of the WFD and SDGs, the current paragraphs dive too deeply into policy details that could be summarized more concisely. For example, instead of quoting each SDG target verbatim, paraphrase the key ideas and focus on how they relate to citizen science.
3. Figures 1-3 are mentioned but not clearly described how they support the narrative. If figures are mentioned this early, a short description of their relevance would help.
4. After outlining the context, the introduction should clearly state what’s missing in the current literature or practice that this review addresses. Right now, that gap is implied but not explicitly stated.
5. While the text lists various benefits and challenges of citizen science, the flow feels fragmented. Consider organizing the introduction around 2–3 key thematic pillars (e.g., why citizen science matters, how it aligns with current frameworks, what needs to be improved) to help guide the reader through the argument.
6. The last paragraph attempts to outline the aim of the piece but remains vague. Phrases like “intended as an open letter” and “based on an assessment of available information” could be replaced with a sharper and more confident statement of what the article seeks to do and why it matters now.
7. Some key claims—particularly those about the benefits of citizen science, global freshwater stressors, and monitoring gaps—would be stronger with additional citations.

State of art

1. The authors give a general idea of how the literature search was conducted, but the term “snowball-like quest” feels a bit vague. Including more detail—like the specific keyword combinations, any time frame used, or how the search was structured—would make the process clearer.
2. The paper mentions that 295 articles were initially identified and 85 selected for further analysis, but the criteria for narrowing this list down could be better explained. It would help to briefly describe how papers were screened or what counted as thematically relevant.

The backbone of citizen science

 Approaches, parameters and data quality

1. The point about citizen science data being 70–90% as accurate as lab values is made twice almost word-for-word. 
2. The section touches on data validation methods, which is really important, but it would help to be a bit more specific. For example, how many of the studies actually used side-by-side comparisons or statistical checks?

Quality of the publication analyzed

1. The quality assessment is based on subjective judgments by a small internal team, which may introduce bias. It would be helpful to either involve a more diverse group or acknowledge this as a limitation more explicitly.

Characteristics of project duration in citizen science

1. The title suggests a focus on project duration, but most of the section talks about data quality and policy use. Either the section should focus more on duration, or the title should be adjusted to better match the content.
2. It would be great to include what helps projects last longer or how duration affects outcomes.

Examples of best practices

 Chemical and optical monitoring

1. Several parts of this section, especially the first two paragraphs, repeat background information already covered in the introduction (e.g., global water quality challenges, the WFD and SDG frameworks). 
2. The title “Chemical and optical monitoring” suggests a technical focus, but much of the content discusses broader background and policy issues already covered in the introduction. Consider adjusting the title or refocusing the content to better match.

The challenges ahead

1. This section raises important points about monitoring gaps and the potential for collaboration with citizen science to address them. However, to strengthen its contribution to the overall review, I suggest narrowing the focus to emphasize actionable recommendations and removing repeated background information covered earlier in the paper. 

Navigating the way forward

 This section touches on many important points, but it lacks focus. It jumps between citizen engagement, river rights, education, and data validation without a clear thread. A more concise and structured approach would help clarify the main message and improve its impact.

Where applicable, are recommendations and next steps explained clearly for others to follow? (Please consider whether others in the research community would be able to implement guidelines or recommendations and/or constructively engage in the debate)

Partly

Does the article adequately reference differing views and opinions?

Partly

Are all factual statements correct, and are statements and arguments made adequately supported by citations?

Yes

Is the rationale for the Open Letter provided in sufficient detail? (Please consider whether existing challenges in the field are outlined clearly and whether the purpose of the letter is explained)

Partly

Is the Open Letter written in accessible language? (Please consider whether all subject-specific terms, concepts and abbreviations are explained)

Yes

Reviewer Expertise:

Citizen Science, Hydrology, Water Monitoring

I confirm that I have read this submission and believe that I have an appropriate level of expertise to state that I do not consider it to be of an acceptable scientific standard, for reasons outlined above.

# Reviewer report: Reviewer response for version 2

**DOI:** 10.21956/openreseurope.21644.r54765

**Published:** 2025-06-09

**Authors:** Ahmet Erkan Kideys

**Affiliations:** 1 Middle East Technical University, Çankaya, Turkey

Authors were complied with the comments and questions I raised in my review. So, I have no further comments and therefore the manuscript is acceptable from my point of view. Thank you

Reviewer Expertise:

marine ecology, citizen science, plastics pollution,

I confirm that I have read this submission and believe that I have an appropriate level of expertise to confirm that it is of an acceptable scientific standard.

# Reviewer report: Reviewer response for version 1

**DOI:** 10.21956/openreseurope.20736.r51327

**Published:** 2025-03-05

**Authors:** Ahmet Erkan Kideys

**Affiliations:** 1 Middle East Technical University, Çankaya, Turkey

- The subject of the paper is highly relevant for enhancing the impact of citizen science from multiple perspectives. It also includes an extensive literature review. However, the **problem statement or hypothesis is not clearly defined at the beginning**. Adding a dedicated subsection on **problem definition** would improve the structure and clarity. The **Conclusions** section should be revised to provide clearer insights, preferably using **bulleted points** to emphasize strategies for **incentivizing** participation in citizen science. This will enhance readability and ensure key takeaways are effectively communicated. I believe the manuscript will be suitable for indexing if the reviewer comments are appropriately addressed.
- **Abstract:** The abstract should be enriched with more concrete findings to enhance clarity and impact.
- **Figure 1:** The resolution of the text and numbers is poor, making them difficult to read. Please improve the quality for better readability.
- **Table 1:** It is unclear whether the keyword **"citizen science"** was specifically used in the search. Please clarify this.
- **Figures 7 and 8:** The relevance of these publications to the **citizen science** subject should be explicitly highlighted for better context.
- The **"Backbones of Citizen Science"** section should incorporate the **10 Principles of Citizen Science** for a more comprehensive discussion.
- In **Figure 10**, a clear explanation is needed as to why **plastic/microplastics** were excluded from this study.
- What type of **invasive species** is *Trachemys scripta*? Please provide clarification.
- **"Pescadores de Plastic (Spain, 2019–2023 https://mon.uvic.cat/pescadors-de-plastic/)"** should be introduced as a new paragraph for better readability and emphasis.
- Examples of best practices would be more appropriate to include as an Appendix.

Is the review written in accessible language?

Yes

Are all factual statements correct and adequately supported by citations?

Yes

Are the conclusions drawn appropriate in the context of the current research literature?

Partly

Is the topic of the review discussed comprehensively in the context of the current literature?

Yes

Reviewer Expertise:

marine ecology, citizen science, plastics pollution,

I confirm that I have read this submission and believe that I have an appropriate level of expertise to confirm that it is of an acceptable scientific standard, however I have significant reservations, as outlined above.

# Response

**Published:** 2025-04-22

**Authors:** Luisa GALGANI

**Affiliations:** University of Siena Department of Biotechnology Chemistry and Pharmacy, Siena, Tuscany, Italy

Dear Editorial Board and Reviewers, 

 We have thoroughly revised our manuscript ‘The role of citizen science within WFD and SDGs, and how to incentivize the collaboration with environmental regulators’, according to the comments received from the reviewers, whom we thank again, comments that we found extremely useful. In lights of the suggestions and the modifications, we would like to change the type of article to “ **Open Letter**” as it best reflects the scope and the structure of the manuscript, and its original intent. We have changed the figures and explained the selection of publications in a flowchart (Figure 4) as suggested. We understand that the manuscript in its first submitted form was lacking important information to be considered a review article. However, we have performed a selection of publications based on thematic keywords, that we explain better in the revised text. The publications screened and for which we report some information are 85 and address citizen science to some extent. From these 85, we sub-selected 34 as the ones that clearly report citizen science generated data and these have been analysed in more details. We have thoroughly revised the text and its sections following the first reviewer's suggestions. The structure of the manuscript is slightly different now. We hope that you find the new version of the manuscript a suitable contribution to Open Research Europe and we are confident that this work will be useful to the journal readers as well as to different stakeholders, including citizens, regulatory agencies and policy makers. Specific comments are addressed below, point-by-point. Our response is in red. We thank Dr. Kideys for his insightful review of the manuscript. We tried to address all queries in the point-by-point reply below. The subject of the paper is highly relevant for enhancing the impact of citizen science from multiple perspectives. It also includes an extensive literature review. However, the **problem statement or hypothesis is not clearly defined at the beginning**. Adding a dedicated subsection on **problem definition** would improve the structure and clarity. The **Conclusions** section should be revised to provide clearer insights, preferably using **bulleted points** to emphasize strategies for **incentivizing** participation in citizen science. This will enhance readability and ensure key takeaways are effectively communicated. I believe the manuscript will be suitable for indexing if the reviewer comments are appropriately addressed. We thank Dr. Kideys for this suggestion. We have revised the introduction, and we hope that the focus of the manuscript is clearer now. We have also thoroughly revised the whole structure of the text from the section “The Backbones of Citizen Science”.  

- **Abstract:** The abstract should be enriched with more concrete findings to enhance clarity and impact.

We have modified the abstract following Dr. Kideys’ suggestions.

  

- **Figure 1:** The resolution of the text and numbers is poor, making them difficult to read. Please improve the quality for better readability.

We thank the reviewer for pointing this out. This image has been taken from the WISE European Environment Agency and we can ask the ORE editorial production to make it larger for better readability.  

- **Table 1:** It is unclear whether the keyword **"citizen science"** was specifically used in the search. Please clarify this.

The text has been revised to address all reviewers’ comments. We hope it is now clearer.

  

- **Figures 7 and 8:** The relevance of these publications to the **citizen science** subject should be explicitly highlighted for better context.

The text has been revised to address all reviewers’ comments. We have revised the list of publications and explained why they were chosen. Also, graphs have been modified. We hope it is now clearer.

  

- The **"Backbones of Citizen Science"** section should incorporate the **10 Principles of Citizen Science** for a more comprehensive discussion.

We have introduced the topic in the new structure of the text provided.

 Thank you for pointing out the lack of clearness concerning the use of “citizen science” as a search word, we have addressed this the new “state of the art” section.  

- In **Figure 10**, a clear explanation is needed as to why **plastic/microplastics** were excluded from this study.

We thank the reviewer for pointing this out. We have included studies looking at plastics and microplastics. However, these parameters are not currently included in the WFD monitoring standard parameters. We suggest that these should be added as they are parameters easily monitored with the help of citizen science.  

- What type of **invasive species** is *Trachemys scripta*? Please provide clarification.

This species is an invasive freshwater turtle also known as the “turtle with the yellow ears”. Its preferred habitats are lakes, ponds and rivers with slow and muddy waters and plenty of aquatic plants. It originates from North-east Mexico and South-east United States. It is present as invasive species in several European countries among which France, Spain, Germany and Italy. We have added more information in the text.

  

- **"Pescadores de Plastic (Spain, 2019–2023** **https://mon.uvic.cat/pescadors-de-plastic/** **)"** should be introduced as a new paragraph for better readability and emphasis.

We thank the reviewer for noticing this. Indeed, this has been overlooked during the pdf finalization process. We will make sure it will be a new paragraph in the new version.  

- Examples of best practices would be more appropriate to include as an Appendix.

We thank the reviewer for the comment, which is also a point raised by both reviewers. These examples are supporting the paper and per ORE guidelines unfortunately they cannot be placed in an appendix or supplementary information. The only possibility would be an external database (as Zenodo), but this would infer on the overall content of the manuscript, so we believe the best option is to leave them in a distinct section of the manuscript itself.

# Reviewer report: Reviewer response for version 1

**DOI:** 10.21956/openreseurope.20736.r51326

**Published:** 2025-03-03

**Authors:** Laura Verbrugge

**Affiliations:** 1 Independent researcher, Espoo, Finland

Thank you for the opportunity to read this paper. It’s aim to review literature on citizen science in a water quality monitoring context is timely as many papers on this topic have been published in the last years. The manuscript has some severe flaws in its goal setting and methodology (see point 1 and 2) that need to be addressed before it can make a contribution to the scientific literature.

 1) In my view, the current paper lacks a clear focus or goal. It is presented as both a literature review and a policy draft which are two different things. I have read the paper as a review as this was indicated in the ‘article type’. Often, it was not clear to me if a statement was a result from the literature review or a more general statement or discussion as this was not clarified. The current paper does not meet the requirements of a literature review, that needs a clear goal, suitable methods to reach this goal, and presentation of the results that answer specific questions. The current paper takes many side paths that are a distraction. A well-defined aim and research questions are needed to fix this issue. Currently, the aim is

- Exploring the state of the art in citizen science-based projects monitoring water quality (-> OK this defines your search for papers using these key terms and synonyms)
- Identifying challenges and opportunities and the potentials for collaboration with national Environmental Agencies and citizens to ensure longer term commitment (-> this could be phrased more specific; what are looking for in the papers? Write specific questions or sentences on how you searched the paper to find this information. For example, which methodology was used for monitoring? What are motivations/drivers and barriers for volunteers? How do these topics link to collaboration with agencies? These are interesting questions, but you never really pose them)
- Providing suggestions to overcome hurdles in upcoming research programmes and activities (-> idem how did you deduct these from the papers?)
- The paper also presents results from a ‘quality assessment’. This should also be included as an aim of this study if those results are presented.

2) The methodology lacks critical information on the literature review protocol. The methods need to be described in such a way that it could be replicated by someone else. The following information needs to be added/ changed:

- Rationale for choosing key words; in my opinion the list of keywords is rather short and arbitrary. What makes you think that these key words captured all relevant available papers? Can you provide evidence for this?
- A description of inclusion and/or exclusion criteria for selecting papers (e.g. you included only studies that reported citizen-science generated data).
- Often the selection process of a literature review is presented as a flow chart starting from the first search results and reporting numbers that meet the criteria, until you reach the final number of papers that was selected (in your case 47). Can you include such as flow chart?
- If you made this selection of papers, why are you reporting results from the screened publications that were not included (end of page 9)?
- Which questions did you aim to answer by reading these papers?
- List of key information etc. that you collected for each paper (this could be added as an appendix) and explanation why this is relevant.

3) Figures need to have a clear purpose linked to the study, e.g. presenting results in a graph or a visual of your conceptual approach or methodology. Many of the Figures in the current paper do not have such a purpose and should be left out in my opinion (e.g. Figures 1 to 6). The remaining Figures need to have clear captions so that it is clear what is presented (is it a result from the literature review?) or what the source is. See more detailed comments in the PDF that I have shared below at the end of the review*.

 4) Overall, in my opinion, the most interesting part of the paper is the results section starting from Backbones. I would recommend to first present quantitative results linked to your research questions (methods, accuracy/validations, type of collaboration etc.), and then a more qualitative review of the challenges, opportunities and best practices (with a clear focus on your research questions).

 5) The examples at the end are nice but do not contribute directly to the goal of the study. In my opinion these could be placed in an appendix instead.

 *I made specific comments in the PDF file. I would like the authors to refer to some detailed comments in the PDF here - https://s3-eu-west-1.amazonaws.com/openreseurope/linked/247605.Review_detailed_comments.pdf

Is the review written in accessible language?

Yes

Are all factual statements correct and adequately supported by citations?

Partly

Are the conclusions drawn appropriate in the context of the current research literature?

Partly

Is the topic of the review discussed comprehensively in the context of the current literature?

Partly

Reviewer Expertise:

citizen science, environmental management

I confirm that I have read this submission and believe that I have an appropriate level of expertise to state that I do not consider it to be of an acceptable scientific standard, for reasons outlined above.

# Response

**Published:** 2025-04-22

**Authors:** Luisa GALGANI

**Affiliations:** University of Siena Department of Biotechnology Chemistry and Pharmacy, Siena, Tuscany, Italy

We have thoroughly revised our manuscript ‘The role of citizen science within WFD and SDGs, and how to incentivize the collaboration with environmental regulators’, according to the comments received from the reviewers, whom we thank again, comments that we found extremely useful. In lights of the suggestions and the modifications, we would like to change the type of article to “ **Open Letter**” as it best reflects the scope and the structure of the manuscript, and its original intent. We have changed the figures and explained the selection of publications in a flowchart (Figure 4) as suggested.

 We understand that the manuscript in its first submitted form was lacking important information to be considered a review article. However, we have performed a selection of publications based on thematic keywords, that we explain better in the revised text.

 The publications screened and for which we report some information are 85 and address citizen science to some extent. From these 85, we sub-selected 34 as the ones that clearly report citizen science generated data and these have been analysed in more details. We have thoroughly revised the text and its sections following the first reviewer's suggestions. The structure of the manuscript is slightly different now.

  We hope that you find the new version of the manuscript a suitable contribution to Open Research Europe and we are confident that this work will be useful to the journal readers as well as to different stakeholders, including citizens, regulatory agencies and policy makers. Specific comments are addressed below, point-by-point. Our response is in red.

**Reviewer 1** Thank you for the opportunity to read this paper. It’s aim to review literature on citizen science in a water quality monitoring context is timely as many papers on this topic have been published in the last years. The manuscript has some severe flaws in its goal setting and methodology (see point 1 and 2) that need to be addressed before it can make a contribution to the scientific literature. We are grateful to Dr. Verbrugge for her insightful suggestions and the thorough review of our manuscript. We are happy to provide the amendments requested.

 1) In my view, the current paper lacks a clear focus or goal. It is presented as both a literature review and a policy draft which are two different things. I have read the paper as a review as this was indicated in the ‘article type’. Often, it was not clear to me if a statement was a result from the literature review or a more general statement or discussion as this was not clarified. The current paper does not meet the requirements of a literature review, that needs a clear goal, suitable methods to reach this goal, and presentation of the results that answer specific questions. The current paper takes many side paths that are a distraction. A well-defined aim and research questions are needed to fix this issue. Currently, the aim is

- Exploring the state of the art in citizen science-based projects monitoring water quality (-> OK this defines your search for papers using these key terms and synonyms)
- Identifying challenges and opportunities and the potentials for collaboration with national Environmental Agencies and citizens to ensure longer term commitment (-> this could be phrased more specific; what are looking for in the papers? Write specific questions or sentences on how you searched the paper to find this information. For example, which methodology was used for monitoring? What are motivations/drivers and barriers for volunteers? How do these topics link to collaboration with agencies? These are interesting questions, but you never really pose them)
- Providing suggestions to overcome hurdles in upcoming research programmes and activities (-> idem how did you deduct these from the papers?)
- The paper also presents results from a ‘quality assessment’. This should also be included as an aim of this study if those results are presented.

We thank the reviewer for pointing this out. We agree with the analysis; our manuscript would best fit the category of Open Letter since the data presented are only related to a small number of studies and it is originally meant as a white paper. With this in mind, we addressed the questions raised in the previous points in the manuscript. We have included an introduction that states the problem, and we hope the focus is now much clearer. We have thoroughly revised the text and its structure and re-formulated suggestions for the future based on the issues identified in the publications screened.

 2) The methodology lacks critical information on the literature review protocol. The methods need to be described in such a way that it could be replicated by someone else. The following information needs to be added/ changed:

- Rationale for choosing key words; in my opinion the list of keywords is rather short and arbitrary. What makes you think that these key words captured all relevant available papers? Can you provide evidence for this?
- A description of inclusion and/or exclusion criteria for selecting papers (e.g. you included only studies that reported citizen-science generated data).
- Often the selection process of a literature review is presented as a flow chart starting from the first search results and reporting numbers that meet the criteria, until you reach the final number of papers that was selected (in your case 47). Can you include such as flow chart?
- If you made this selection of papers, why are you reporting results from the screened publications that were not included (end of page 9)?
- Which questions did you aim to answer by reading these papers?
- List of key information etc. that you collected for each paper (this could be added as an appendix) and explanation why this is relevant.

Thank you for this comment. We have provided the data underlying the publication which is an Excel file available on Zenodo. The link is here https://zenodo.org/records/14633986 and it is also stated in the data availability section of the manuscript. It contains key information about the papers individuated for the analysis.

 We agree that the rationale for the selection of keywords was not clearly stated, we have addressed this issue in the manuscript in the new text. As this article was not intended to be a comprehensive literature review, we acknowledge that there may be some important publications that we missed. A description of the selection criteria has been provided in the manuscript in the section “State of the art”, detailing how we selected publications based on their following of ECSA’s 10 principles of citizen science. Furthermore we highlighted which principles were the most followed, and why the remaining publications were not selected but still reported. We hope this amended section clarifies the process we followed. We have provided a new figure (Figure 4) with the flowchart for the publications selection process. Figures have changed and are now renumbered. We have performed a re-analysis of the publications, identifying 85 that deal with citizen science and address citizen science to some extent. These 85 have been thematically categorized, as described in the text. From these 85, 34 have been included in a sub-selection and analyzed in details because these clearly report citizen generated data. 

 3) Figures need to have a clear purpose linked to the study, e.g. presenting results in a graph or a visual of your conceptual approach or methodology. Many of the Figures in the current paper do not have such a purpose and should be left out in my opinion (e.g. Figures 1 to 6). The remaining Figures need to have clear captions so that it is clear what is presented (is it a result from the literature review?) or what the source is. See more detailed comments in the PDF that I have shared below at the end of the review*. Thank you for the comment. We have removed some figures. However, in light of the new scope of the manuscript as an open letter, some figures should be kept.  In particular, we believe the infographics are useful to express concepts that are hindered in the text and would go as additional text if not included in the figure. In our opinion, these infographics are useful as they help memorizing some key messages to serve the purpose of the present publication. The figures’ numbers have changed. Please see the revised version for the new order.

 4) Overall, in my opinion, the most interesting part of the paper is the results section starting from Backbones. I would recommend to first present quantitative results linked to your research questions (methods, accuracy/validations, type of collaboration etc.), and then a more qualitative review of the challenges, opportunities and best practices (with a clear focus on your research questions). We thank Dr. Verbrugge for this suggestion. We have thoroughly revised the structure of the whole text starting from the section “The backbones of Citizen Science” and we hope the text has now improved.

 5) The examples at the end are nice but do not contribute directly to the goal of the study. In my opinion these could be placed in an appendix instead. We thank the reviewer for the comment. These examples are supporting the paper and following ORE guidelines unfortunately they cannot be placed in an appendix or supplementary information. The only possibility would be an external database (as Zenodo), but this would infer on the overall content of the manuscript, so we believe the best option is that they remain as a distinct section of the manuscript itself.

 *I made specific comments in the PDF file. I would like the authors to refer to some detailed comments in the PDF here - https://s3-eu-west-1.amazonaws.com/openreseurope/linked/247605.Review_detailed_comments.pdf We thank Dr. Verbrugge for the detailed review. We have addressed all comments in the pdf. Please see the pdf attached with the revised version.
